# Lipidomic Profiling Reveals the Reducing Lipid Accumulation Effect of Dietary Taurine in Groupers (*Epinephelus coioides*)

**DOI:** 10.3389/fmolb.2021.814318

**Published:** 2021-12-24

**Authors:** Fakai Bai, Xuexi Wang, Xingjian Niu, Guiping Shen, Jidan Ye

**Affiliations:** ^1^ Xiamen Key Laboratory for Feed Quality Testing and Safety Evaluation, Fisheries College of Jimei University, Xiamen, China; ^2^ Key Laboratory of Marine Biotechnology of Fujian Province, Institute of Oceanology, Animal Sciences, Fujian Agriculture and Forestry University, Fuzhou, China; ^3^ Fujian Provincial Key Laboratory of Plasma and Magnetic Resonance, Department of Electronic Science, Xiamen University, Xiamen, China

**Keywords:** taurine, lipidomics, fatty acid composition, positional distribution, *Epinephelus coioides*

## Abstract

A lipidomic analysis was conducted to provide the first detailed overview of lipid molecule profiles in response to dietary lipid and taurine and associations of liver lipid-lowering effects of dietary taurine with lipid molecular species and the positional distributions of fatty acids in the liver of juvenile orange-spotted groupers (*Epinephelus coioides*). The results indicated that the liver was more sensitive to varied dietary lipid and taurine contents than the muscle with regard to lipid molecules. A total of 131 differential lipid molecules (DLMs) were observed in the liver of groupers when dietary taurine was increased from 0 to 1% at 15% lipid, among which all the up and down-regulated DLMs are phospholipids (PLs) and triglycerides (TGs), respectively. The liver content of TGs containing 18:2n-6 attached at the *sn-2* and *sn-3* positions on the glycerol backbone increased with increasing dietary lipid from 10 to 15% but decreased with increasing dietary taurine from 0 to 1%. Therefore, dietary taurine can not only reduce lipid accumulation through decreasing the contents of TGs containing 18:2n-6 at the *sn-2* and *sn-3* positions but also enhance the anti-inflammatory capacity and health status of groupers. This study will also provide a new insight into the function of taurine in farmed fish.

## Introduction

Taurine (2-aminoethanesulfonic acid) is rich in animals. It does not participate in protein synthesis because it contains sulfonic acid groups but no carboxyl groups. Taurine is known to play a wide range of key roles in animal physiology, including functions in bile acid conjugation, immune regulation, osmoregulation, antioxidation, nervous system development, and regeneration (Huxtable, 1992; Salze and Davis, 2015; Wu, 2020; Xu et al., 2020). It appears that taurine has a lipid-lowering effect, as demonstrated by the ability of dietary taurine to reduce the lipid contents of tissues in fish (Salze and Davis, 2015; Hu et al., 2018; Garcia-Organista et al., 2019; Martins et al., 2021). For example, dietary taurine addition resulted in a reduction of lipid content in the whole body, muscle, or liver in California yellowtails (*Seriola dorsalis*) (Garcia-Organista et al., 2019) and rice field eels (*Monopterus albus*) (Hu et al., 2018), whereas an enhancement of lipid contents in the whole body was reported in turbots (*Scophthalmus maximus*) (Qi et al., 2012). However, dietary taurine did not affect lipid content in the liver and muscle but reduced the abundance of individual PLs (phospholipid molecules) and enhanced individual liver ceramides and TGs (triglyceride molecules) in tiger puffers (*Takifugu rubripes*) (Xu et al., 2020). The varied lipid contents in different tissues induced by dietary taurine addition may be related to the changes of lipid molecules (Salze and Davis, 2015), which can be divided into lipids (TGs) and lipoids (cholesterol, cholesterol ester, and PLs) (Koelmel et al., 2017; Lam et al., 2017; Luo et al., 2017).

Among the lipid molecules, TGs play a critical role by acting as the central molecules of the lipid metabolism, and mainly accumulate in hepatic cells (Luo et al., 2017; Peng et al., 2021). PLs are another class of extremely important lipid molecules that constitute the main component of biological membranes and are responsible for a variety of cellular functions (Lordan et al., 2017). According to their molecular structure, PLs can be divided into several categories, mainly including phosphatidylethanolamine, phosphatidylinositol, phosphatidylserine, phosphatidylcholine, and sphingomyelin (Lordan et al., 2017). Recent research has shown that different fatty acids (FAs) are arranged at different positions of the lipid–glycerol backbone of lipid molecules which affects the efficiency of FA hydrolysis, absorption, and utilization in Nile tilapias (*Oreochromis niloticus*) (Liu et al., 2019) and mud crabs (*Scylla paramamosain*) (Wang et al., 2022). The TGs consist of a glycerol backbone to which three acylated FAs are attached (Yuan et al., 2021; Wang et al., 2022). The positions of FA attachment are, respectively, named by stereospecific numbers, *sn-1*, *sn-2*, and *sn-*3 in sequence from top to bottom, and the stereospecific positions of individual FAs are key to determine the fate of the TG metabolism (Hussein et al., 2019; He et al., 2021). Therefore, the location of FAs on the glycerol backbone is of importance for fish to use lipids (Liu et al., 2019). Here, we hypothesize that the reduction of liver lipid accumulation caused by dietary taurine is also linked to shifts of glycerol positional distributions of FAs on the glycerol backbone in fish. However, little is known about the modulation of the lipid metabolism regarding the lipid retention and the positional distribution of FAs of the glycerol backbone in different lipid species in response to dietary taurine.

Lipidomics is a mass spectrometry–based technology, which enables to accurately detect the complete lipid profile, functions, and structures within a tissue; thus, it is a tool to investigate metabolic dysfunction associated with metabolic diseases, such as fatty liver, diabetes, and cardiovascular diseases (Hussein et al., 2019; Liu et al., 2019; He et al., 2021). Nevertheless, there are few reports on the role of lipid molecules in the fish lipid metabolism based on lipidomics. Therefore, in this study, lipidomics was performed to determine how dietary taurine contents influence the lipid composition and the abundance of lipids in the liver and muscle of groupers at normal and higher dietary lipid contents in an attempt to investigate the association of lipid molecule shift with dietary taurine and their modulation of the lipid metabolism in the fish species.

The orange-spotted grouper (*Epinephelus coioides*) is a carnivorous fish species that is becoming an increasingly important farmed fish in the aquaculture industry in Southeast Asian countries, including China (Shapawi et al., 2019; Feng et al., 2020). Previous studies showed that dietary taurine addition can attenuate tissue lipid deposit of groupers (Koven et al., 2016; Lin and Lu, 2020); however, the underlying mechanism involved in the regulation of the lipid metabolism is not fully elucidated (Shen et al., 2019; Bai et al., 2021). As for associations of dietary lipids with the growth of the grouper, when the dietary lipid content was 10%, the fish species obtained ideal growth, but when dietary lipid content continued to increase (from 12 to 14%), the growth rate of the grouper showed a downward trend, accompanied by a marked increase in liver lipid accumulation (Luo et al., 2005). In this study, therefore, we prepared four experimental diets containing two taurine contents (taurine-free and 1% taurine) and two lipid contents (10 and 15%) to investigate the growth rate, tissue lipid contents, and lipidomic profile in response to dietary taurine and lipid contents. Our aims were to determine whether dietary taurine affected the composition and abundance of lipid molecules and the positional distributions of FAs on the glycerol backbone in different lipid species in grouper liver. The present study might provide a novel insight into the roles of taurine in the lipid metabolism in fish.

## Materials and Methods

### Growth Trial

Based upon previous studies with the optimal dietary contents of lipids (10%) and taurine (1%) for the growth of *E. coioides* (Luo et al., 2005; Zhou et al., 2015), a basal diet (10% lipid and taurine-free, D1) was formulated to contain 47% crude protein, 10% crude lipid, and zero taurine addition using fish oil, soy oil, and soy lecithin as the lipid sources, taurine-free casein and gelatin as the protein sources, corn starch as the carbohydrate source, and microcrystalline cellulose as the filler. The addition of blend oil (fish: soy oil = 1:1) or taurine was at the expense of the reduction of microcrystalline cellulose in the basal diets to prepare three isonitrogenous (47% crude protein) diets (10% lipid and 1% taurine, D2; 15% lipid and taurine-free, D3; and 15% lipid and 1% taurine, D4). The ingredients and proximate compositions of the experimental diets are presented in Supplementary Table S1. The experimental diets were manufactured and then stored at −20°C prior to use as previously described (Bai et al., 2021). Juvenile groupers were obtained from a local hatchery (Zhangpu County, Fujian, China). After 3 weeks of acclimation, a total of 360 fish with an initial wet weight of 10.5 ± 0.1 g were randomly distributed into four groups with triplicate 300 L tanks at a stock density of 30 fish per tank, within a recirculating system maintaining a water flow rate of 8 L/min. The feeding and management of fish were described as mentioned previously (Bai et al., 2021). During the feeding period, the water temperature was maintained at 28.0 ± 0.3°C, the dissolved oxygen content was maintained at 6.2 ± 0.22 mg/L, and the ammonia nitrogen content was maintained at 0.19 ± 0.03 mg/L.

### Growth Rate Calculation and Tissue Composition Analysis

At the end of the feeding trial, the fish were batch-weighted by the tank to determine the growth rate on a wet weight basis after 24 h starvation. Five fish were randomly sampled from each tank and sacrificed with an overdose of MS-222 solution (tricaine methanesulfonate, Sigma-Aldrich Shanghai Trading Co. Ltd., Shanghai, China). The liver and dorsal muscles were then aseptically removed and pooled into one tube for each tank and stored at −80°C for the subsequent determination of biochemical components.

The proximate composition of ingredients, diets, and tissues was determined according to standard methods (Bai et al., 2021). Total lipids from liver and muscle samples were extracted with chloroform–methanol (2:1, vol/vol) solution according to the Folch protocol (Folch et al., 1957) and determined gravimetrically after drying a 5 ml aliquot under nitrogen. For taurine determination (Aragão et al., 2014), the samples of diets were hydrolyzed in 6 mol/L HCl at 116°C for 22 h in nitrogen-flushed glass vials and centrifuged at 1,500 *g* for 15 min at 4°C, and the supernatant was collected and applied to an automatic AA analyzer (Hitachi L8900, Tokyo, Japan).

### Lipid Analysis and Identification

In the present study, 1% dietary taurine addition caused a decrease in liver lipid content and had no significant effect on muscle lipid content vs. taurine-free diets. Based upon the results, D1 (10% lipid and taurine-free), D2 (10% lipid and 1% taurine), D3 (15% lipid and taurine-free), and D4 (15% lipid and 1% taurine) were selected to make a pairwise comparison of the results. The pooled liver and muscle samples of five fish from each tank were used for lipid analysis (three replicates for each dietary treatment). Lipids were extracted according to the methyl tert-butyl ether (MTBE) method (Matyash et al., 2008). Briefly, approximately 30 mg of the sample was weighed and transferred to a 2 ml centrifuge tube pre-equipped with an appropriate number of magnetic beads; 200 μL of water was added at 4°C, and the mixture was put into liquid nitrogen to flash freeze 5 s and homogenized. Then, 240 μL of ice-cold methanol was added and vortex-mixed; 800 µL of MTBE was added, and the mixture was ultrasonically shaken for 20 min at 4°C, followed by standing still for 30 min at 25°C. The solution was centrifuged at 14,000 *g* for 15 min at 10°C, and the upper (organic) phase was obtained and dried under nitrogen and stored at −80°C.

The samples were separated using a UHPLC Nexera LC-30A system (Shimadzu Corporation, Tokyo, Japan) and placed in a 10°C automatic sampler with a column temperature of 45°C and a flow rate of 300 μL/min. Solvent A was 10 mM ammonium formate acetonitrile aqueous solution (acetonitrile: water = 6:4, v/v); solvent B was 10 mM ammonium formate acetonitrile isopropanol solution (acetonitrile: isopropanol = 1:9, v/v). The elution gradient was performed as follows: from 0 to 2 min, solvent B was held at 30%; from 2 to 25 min, solvent B was linearly increased to 100%; and from 25 to 35 min, solvent B was maintained at 30%.

Mass spectra were recorded using a Q-Exactive Plus (Thermo Fisher Scientific, CA, United States) in positive and negative modes. ESI parameters were optimized and preset for all measurements as follows: positive: heater temperature 300°C, sheath gas flow rate 45 arb, aux gas flow rate 15 arb, sweep gas flow rate 1 arb, spray voltage 3.0 KV, capillary temperature 350°C, S-Lens RF level 50%; MS1 scan ranges: 200–1800; negative: heater temperature 300°C, sheath gas flow rate 45 arb, aux gas flow rate 15 arb, sweep gas flow rate 1 arb, spray voltage 2.5 KV, capillary temperature 350°C, S-Lens RF level 60%. The mass charge ratio of lipid molecules to lipid fragments was obtained as follows: 10 fragment profiles (MS2 scan, HCD) were obtained after each full scan. MS1 has a resolution of 70,000 at M/Z 200, and MS1 has 17,500 at M/Z 200.

LipidSearch software v4.1.16 (Thermo Fisher Scientific™, CA, United States) was used for lipid identification (secondary identification), peak identification, peak extraction, peak alignment, and quantitative processing. Parameter settings were as follows: precursor tolerance, 5.0 ppm; product tolerance, 5.0 ppm; product ion threshold, 5%.

### Statistical Analysis

Data were presented as the mean and standard errors of the mean (SEM). Data were first analyzed by using one-way analysis of variance (ANOVA) to assess differences among all four treatments. Two-way ANOVA was then used to test the treatment effects of dietary taurine and lipid contents on growth rate and liver and muscle lipid contents. The Student–Neuman–Keuls multiple comparison test was determined after confirming the normality and homogeneity of variance using the Kolmogorov–Smirnov test and Levene’s test in SPSS Statistics 22.0 (SPSS, Michigan Avenue, Chicago, IL, United States). The position distribution of FAs in TGs, PCs, and PEs was assessed by using Student’s t-test. GraphPad Prism 9.0 (San Diego, CA, United States) software was used for the processing of the histogram. Data expressed as percentages or ratios were subjected to data transformation before statistical analysis. A *p*-value < 0.05 was considered statistically significant.

The raw data were normalized and imported into SIMCA-P 14.0 (Umetrics, Umea, Sweden) for analysis and visualization by multivariate statistical methods, including principal component analysis (PCA) and orthogonal partial least squares discriminant analysis (OPLS-DA) (Chen et al., 2021). PCA was performed by using a mean-centered approach in order to identify intrinsic trends and obvious outliers within the data set. A more sophisticated discriminant technique, OPLS-DA, was further applied under a Pareto scaling pattern to achieve global profile separation between the different dietary groups through maximizing the inter-group variance and thereby obtaining specific differential metabolites. The quality of the model was described by the cross-validation parameter Q^2^, indicating the predictability of the model, and *R* ^2^, indicating the total explained variation for the data. The major differences in lipid molecules between pairwise comparison groups were determined based on the variable importance for the projection (VIP). In this experiment, VIP > 1 and *p*-value < 0.05 were used as the screening standard and the significant differences between each group, respectively.

## Results

### Growth Performance and Lipid Contents of Tissues

**FIGURE 1:**
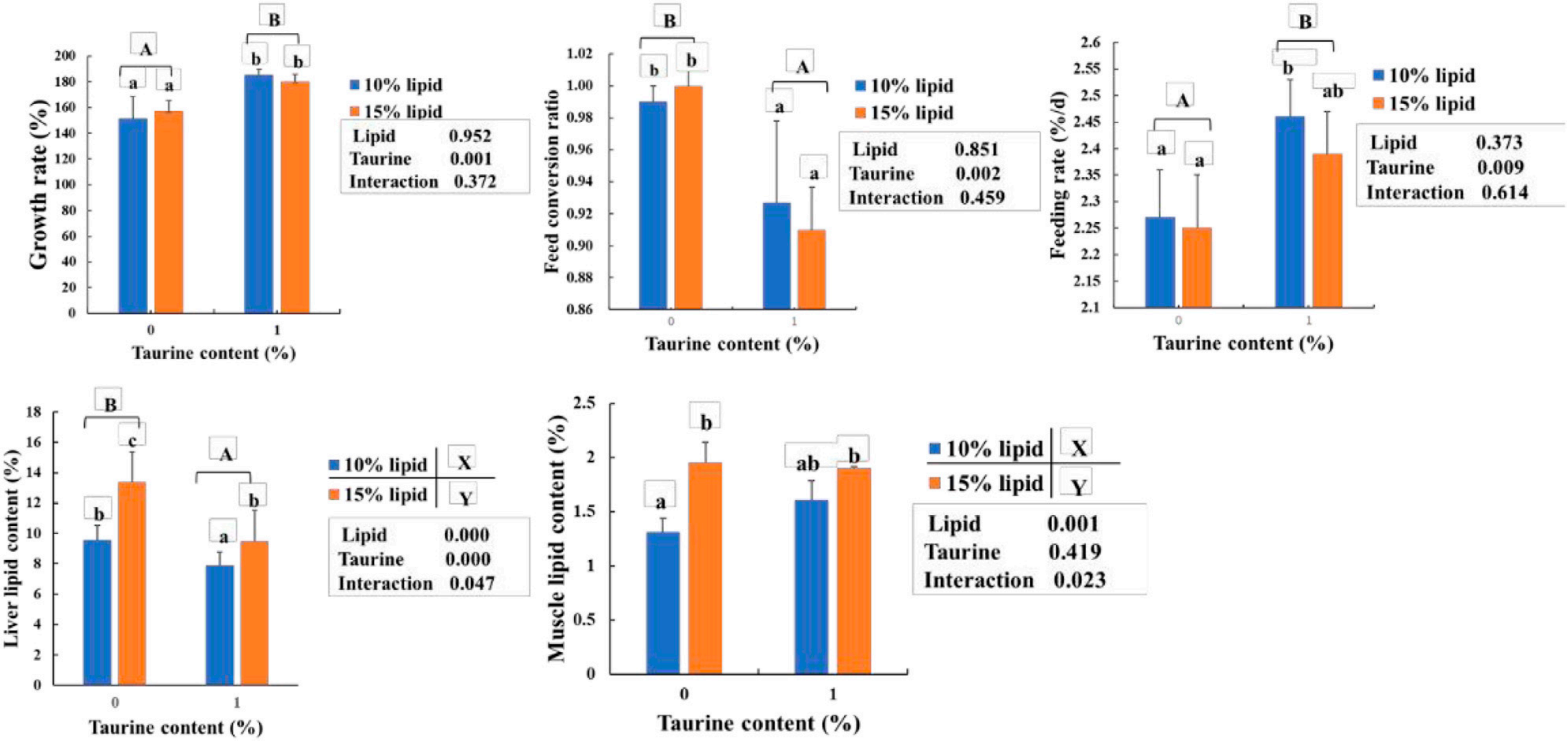
Effects of dietary lipid and taurine contents on growth rate **(A)**, liver **(B)**, and muscle **(C)** lipid contents of groupers in a 56 day feeding period. Growth rate (%) = 100 × (final body weight (g/fish) – initial body weight (g/fish))/initial body weight (g/fish). Feed conversion ratio = feed intake (g/fish)/((final body weight (g/fish)—initial body weight (g/fish)). Feeding rate (%/d) = 100 × (feed intake (g/fish)/((final body weight (g/fish) + initial body weight (g/fish))/2×days). Data are presented as the means of three triplicates per dietary treatment (mean ± SEM). Statistical analysis was performed by one- and two-way ANOVA, followed by Student–Neuman–Keuls multiple comparison. Values in the same column with different uppercase or lowercase letter superscripts indicate significant differences (*p* < 0.05), while with the same letter or no letter superscripts indicate no significant difference (*p* > 0.05). Values with different superscripts (X and Y) indicate significant differences (*p* < 0.05) within the lipid diet groups, regardless of dietary taurine content. Values with different superscripts (A and B) indicate significant differences (*p* < 0.05) within taurine diet groups, regardless of dietary lipid content.

As shown in Figure 1, the lipid contents of the liver and muscle in 15% lipid diets were higher (*p* < 0.05) than that in 10% lipid diets, regardless of the dietary taurine content. The growth rate and feeding rate were higher, and the feed conversion ratio and liver lipid contents in the taurine diets were significantly lower (*p* < 0.05) than that in the taurine-free diet, regardless of dietary lipid content. However, the muscle lipid content was not influenced by dietary taurine. Furthermore, significant (*p* < 0.01) interactions between dietary taurine and lipid contents were portrayed in the muscle and liver lipids.

### Lipid Composition in the Liver and Muscle

**FIGURE 2:**
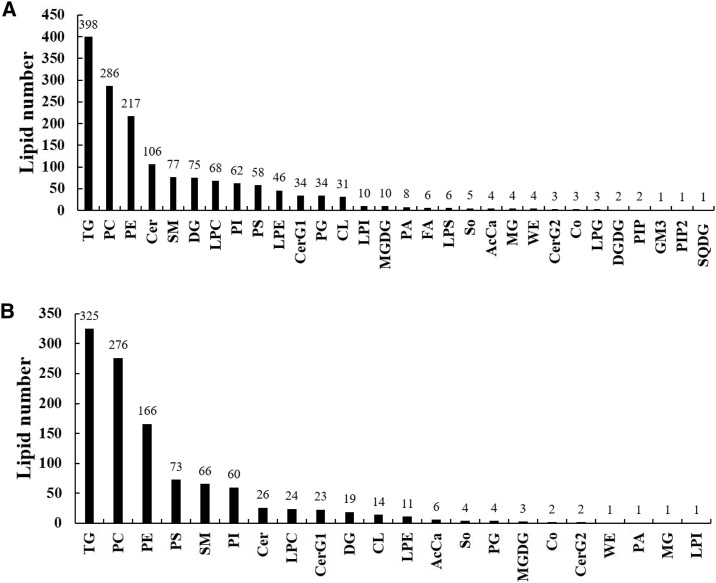
Lipid classes and molecules in the liver **(A)** and muscle **(B)** of groupers fed with different diets in a 56 day feeding period. TG, triglyceride; PC, phosphatidylcholine; PE, phosphatidylethanolamine; Cer, ceramides; SM, sphingomyelin; DG, diglyceride; LPC, lysophosphatidylcholine; PI, phosphatidylinositol; PS, phosphatidylserine; LPE, lysophosphatidylethanolamine; CerG1, glucosylceramide; PG, phosphatidylglycerol; CL, cardiolipin; LPI, lysophosphatidylinositol; MGDG, monogalactosyldiacylglycerol; PA, phosphatidic acid; FA, fatty acid; LPS, lysophosphatidylserine; So, sphingosine; AcCa, acyl carnitine; MG, monoglyceride; WE, wax exters; CerG2, glucosylceramide; Co, coenzyme; LPG, lysophosphatidylglycerol; DGDG, digalactosyldiacylglycerol; PIP, phosphatidylinositol(4) phosphate; GM3, gangliosides; PIP2, phosphatidylinositol(4,5) bisphosphate; SQDG, sulfoquinovosyldiacylglycerol.

A total of 1,565 lipid molecules in the liver were detected and identified, belonging to 30 lipid classes (Figure 2A). There were 10 classes with more than 40 lipid molecules, which are triglycerides (TGs, 398), phosphatidylcholines (PCs, 286), phosphatidyl ethanolamines (PEs, 217), ceramides (Cers, 106), sphingomyelins (SMs, 77), diacylglycerols (DGs, 75), lysophosphatidylcholines (LPCs, 68), phosphatidylinositols (PIs, 62), phosphatidylserines (PSs, 58), and lysophosphatidyl ethanolamines (LPEs, 46).

A total of 1,108 lipid molecules in the muscle were detected and identified, belonging to 22 lipid classes (Figure 2B). There were six classes with more than 40 lipid molecules, including TGs (325), PCs (276), PEs (166), PSs (73), SMs (66), and PIs (60).

### Lipidomic Multivariate Analysis

**FIGURE 3:**
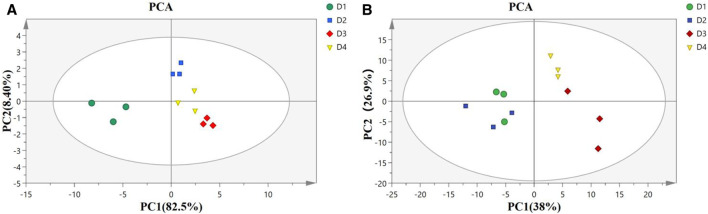
Principal component analysis (PCA) model of the liver **(A)** and muscle **(B)** in groupers fed with different diets in a 56 day feeding period. D1, 10% lipid and taurine-free; D2, 10% lipid and 1% taurine; D3, 15% lipid and taurine-free; and D4, 15% lipid and 1% taurine.

PCA score plot in the liver (Figure 3A) showed well-separated clusters for diet D1 (10% lipid and taurine-free), diet D2 (10% lipid and 1% taurine), diet D3 (15% lipid and taurine-free), and diet D4 (15% lipid and 1% taurine). The first two principal components accounted for 90.9% (PC1: 82.5%; PC2: 8.4%) of the total variance in the data. The result of PCA score plots in the muscle (Figure 3B) showed four general-separated clusters for diets D1–D4, which explained 38 and 26.9% of the total variance in the first and second principal components, respectively. There was a small overlap between the clusters from groups D1 and D2 in the muscle.

**FIGURE 4:**
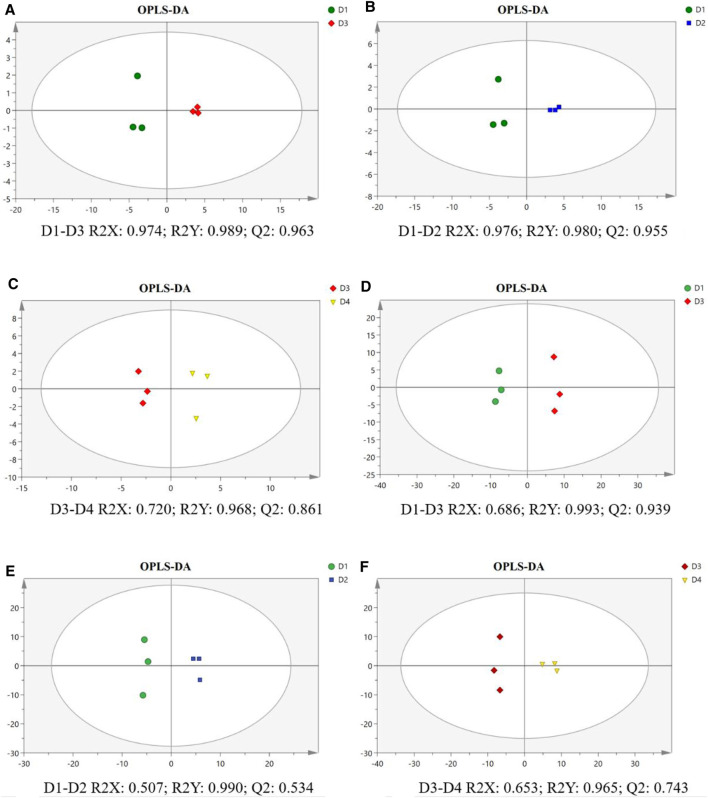
Orthogonal partial least squares discriminant analysis (OPLS-DA) score plot and validation plot of the liver **(A–C)** and muscle **(D–F)** in different groups of groupers. The parameter R2X demonstrates the X variables being explained by the model, R2Y represents the Y variables, and Q2 indicates the prediction ability of the mode. All samples are within 95% confidence intervals. D1, 10% lipid and taurine-free; D2, 10% lipid and 1% taurine; D3, 15% lipid and taurine-free; D4, 15% lipid and 1% taurine.

**FIGURE 5:**
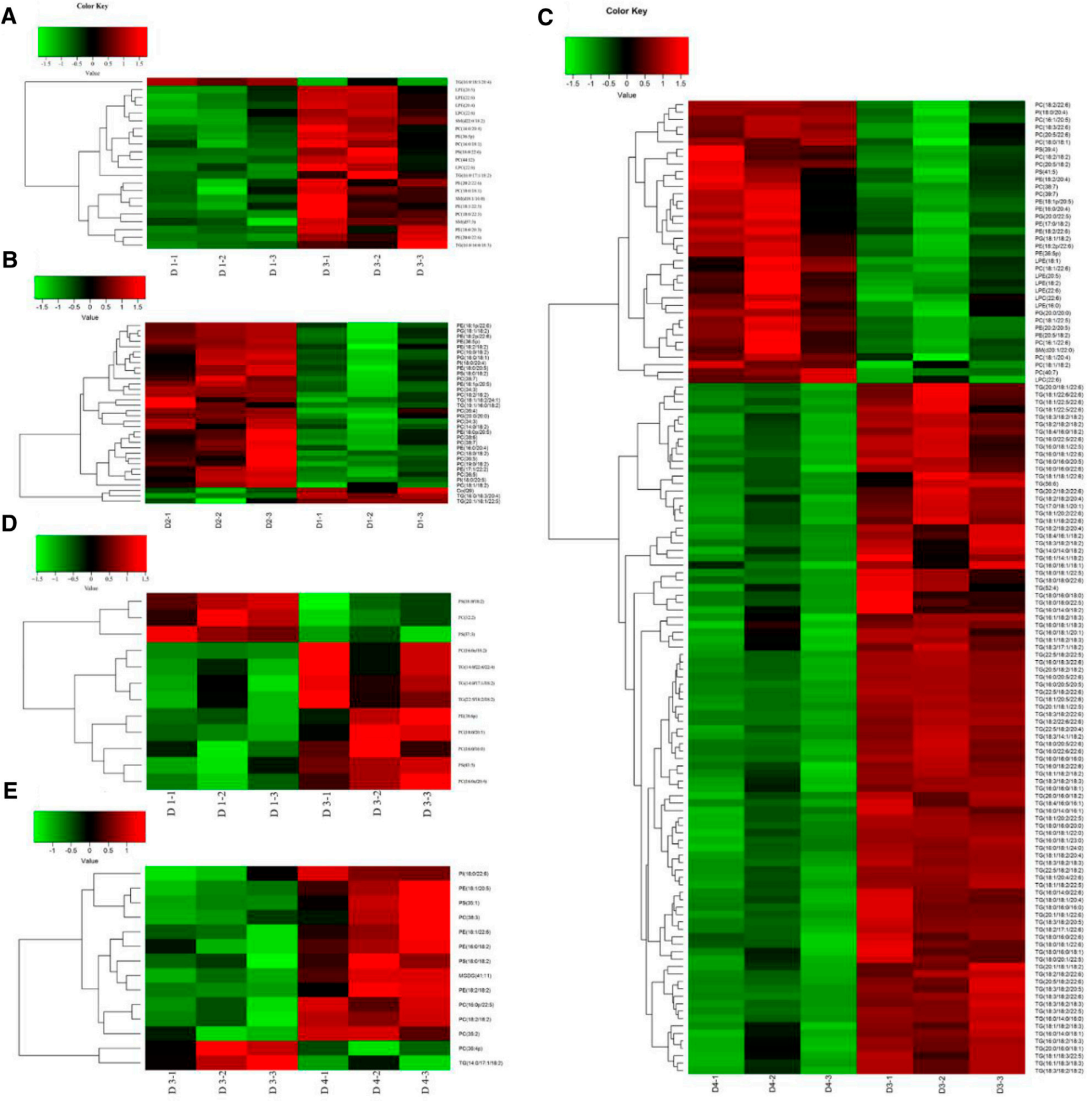
Differential lipid hierarchical clustering in the liver **(A–C)** and muscle **(D–E)** of groupers. **(A)** Hierarchical cluster heatmap (D1–D3); **(B)** hierarchical cluster heatmap (D1–D2); **(C)** hierarchical cluster heatmap (D3–D4); **(D)** hierarchical cluster heatmap (D1–D3); **(E)** hierarchical cluster heatmap (D3–D4). Heatmap of hierarchical clustering analysis for each pairwise comparison group. The horizontal coordinate indicates different experimental groups (three-column replicates belong to one experimental group), and the vertical coordinate indicates different lipid molecules of this group. D1, 10% lipid and taurine-free; D2, 10% lipid and 1% taurine; D3, 15% lipid and taurine-free; D4, 15% lipid and 1% taurine.

As shown in the OPLS-DA score plots, the separations of pairwise comparison groups in the liver (Figures 4A–C) and muscle (Figures 4D–F) showed a suitable predictive ability for data. To further analyze lipidomic data, VIP was used to detect the significance of the difference, and the results are presented in Supplementary Figures S1A–C for the liver and in Supplementary Figures S1D–F for the muscle. The differential lipid molecules (DLMs) are presented in Supplementary Tables S2–S7. We also used hierarchical clustering to display the alterations of DLMs in pairwise comparison groups of the liver (Figures 5A–C) and muscle (Figures 5D–E).

### Lipid Alterations of Pairwise Comparison Groups

The DLMs in the liver are displayed in Figures 5A–C and Supplementary Tables S2–S4. Compared with diet D1, a total of 22 DLMs in the liver were identified in diet D3, of which 21 were upregulated (*p* < 0.05) and only TGs (16:0/18:3/20:4) were downregulated (*p* < 0.05). Compared with diet D1, the dietary taurine addition caused a change in a total of 34 DLMs in the liver in diet D2. The downregulated DLMs were mainly TGs (*p* < 0.05), and the upregulated DLMs were mainly PEs and PCs (*p* < 0.05). Compared with diet D3, the dietary taurine addition caused a change in a total of 131 DLMs in the liver in diet D4, 38 of which were upregulated (*p* < 0.05), mainly including 14 PCs, 9 PEs, 3 phosphatidyl glycerols, and 5 LPEs, and the remaining 93 were downregulated (*p* < 0.05), which were mainly TGs.

The DLMs in the muscle are shown in Figures 5D–F and Supplementary Tables S5–S7. Compared with diet D1, a total of 12 DLMs were identified in the muscle of diet D3. Among them, the contents of PSs (18:0/18:2), PCs (16:1/16:1), and PSs (17:0/20:3) were downregulated (*p* < 0.05), and the rest were upregulated (*p* < 0.05). Compared with diet D1, dietary taurine addition had little effect on muscular lipid molecules in diet D2. Compared with diet D3, dietary taurine addition caused a change in a total of 14 DLMs in diet D4, mainly including 4 PEs, 2 PSs, 5 PCs, and 1 PI, 1 monogalactosyldiacylglycerol, and 1 TG type. Among them, the contents of PCs (18:2/18:2) and TGs (14:0/17:1/18:2) were downregulated (*p* < 0.05) by dietary taurine addition.

### Analysis of the Predominant DLMs in the Liver

**FIGURE 6:**
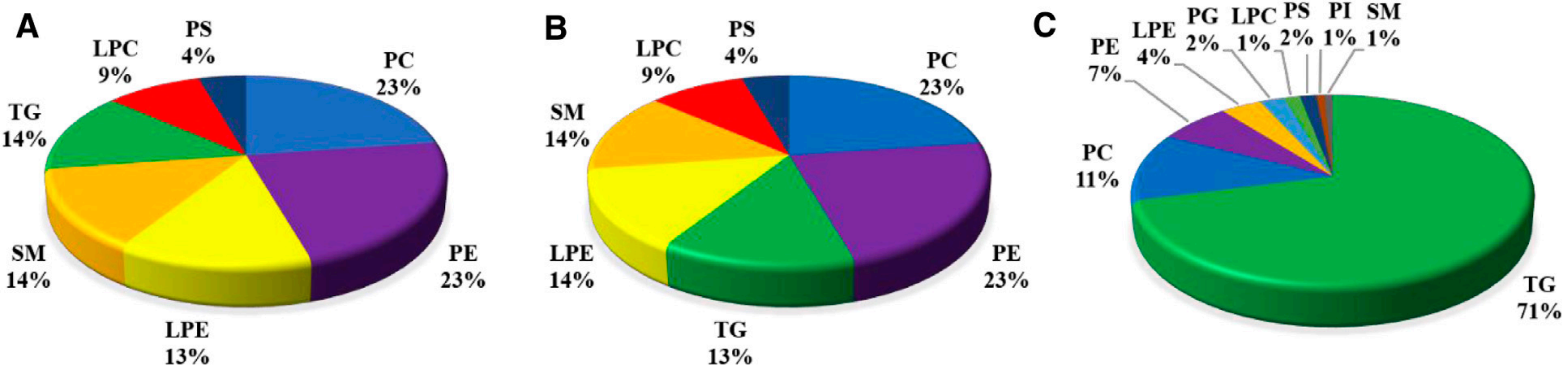
Composition of different lipid molecules (number, percentage) in the liver of groupers in D1 vs.. D3 **(A)**, D1 vs. D2 **(B)**, and D3 vs. D4 **(C)**. D1, 10% lipid and taurine-free; D2, 10% lipid and 1% taurine; D3, 15% lipid and taurine-free; D4, 15% lipid and 1% taurine.

Based upon the abovementioned results, there was a greater number of DLMs in the liver than in the muscle in comparison with diet D3 and diet D4. The liver fatty acid profiles of groupers fed with diet D3 and diet D4 are presented in Supplementary Table S8. As can be seen from this table, feeding diet D3 and diet D4 resulted in distinct changes in the liver contents of some fatty acids. We therefore selected diet D3 and diet D4 as the pairwise comparisons to further determine the variations of the FAs in TGs, PCs, and PEs in the liver in response to dietary taurine. The composition of different lipid metabolites is displayed in Figure 6A–C. Compared with diet D1, 23% PCs, 23% PEs, and 13% TGs in the liver were identified in diet D3. Compared with diet D1, 41% PCs, 26% PEs, and 12% TGs in the liver were identified in diet D2. 71% TGs, 11% PCs, and 7% PEs in the liver were identified in diet D4 compared to diet D3. The main DLMs in all pairwise comparison groups were TGs, PCs, and PEs in the liver.

### The Liver Distribution of Key FAs in TGs, PCs, and PEs

**FIGURE 7:**
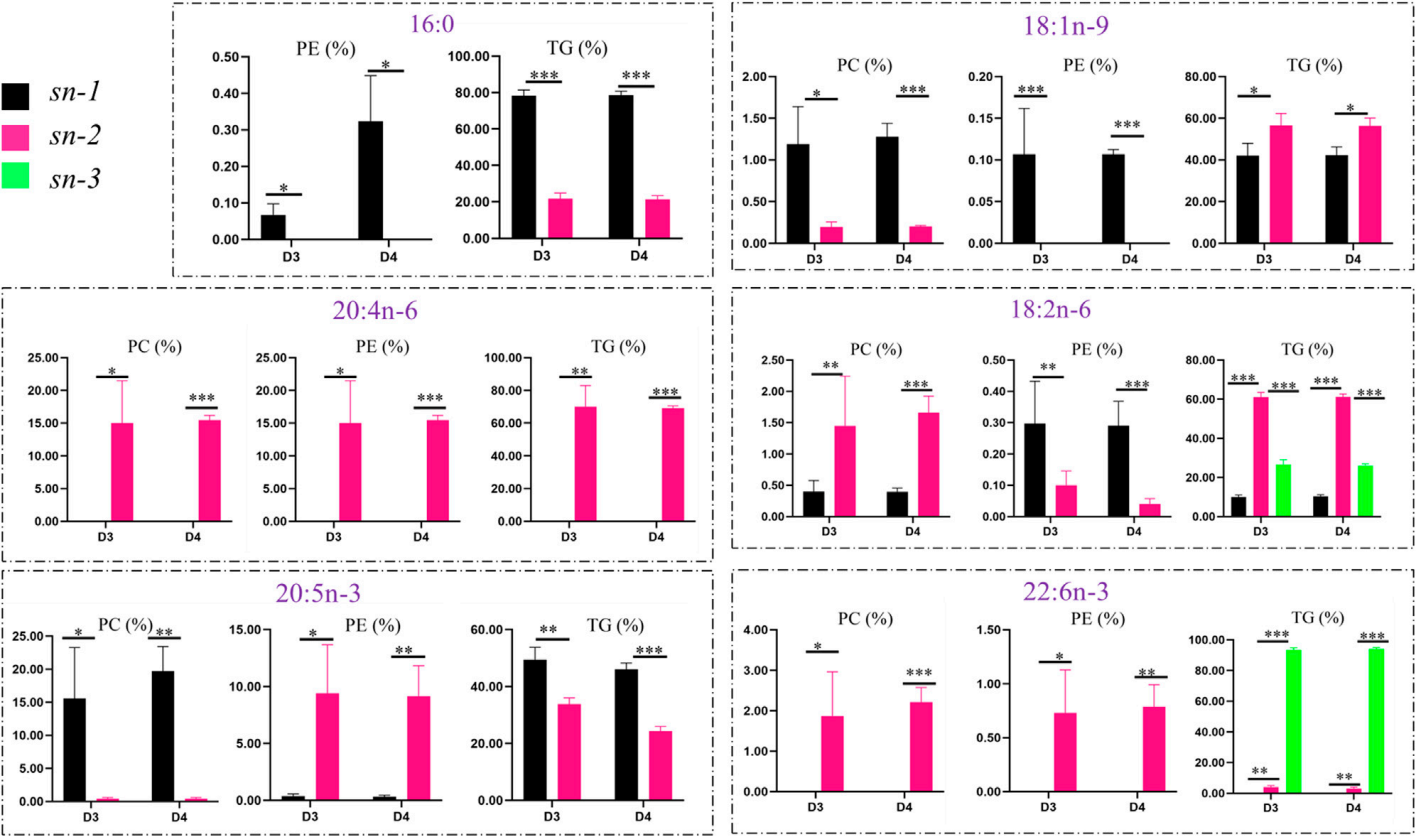
Comparison of positional distributions of key fatty acids in phosphatidylcholine (PC), phosphatidylethanolamine (PE), and triacylglycerol (TG) molecules in the liver of groupers fed with diet D3 and diet D4. The positions of fatty acid attachment on the glycerol backbone of triacylglycerol molecules are, respectively, named by stereospecific numbers, *sn-1*, *sn-2*, and *sn-3* in sequence. Data are presented as the means of three triplicates per dietary treatment (mean ± SEM). Statistical analysis was performed using Student’s t-test. Asterisks (*, **, and ***) represent significant differences with *P* < 0.05, *P* < 0.01, and *P* < 0.001, respectively. D3, 15% lipid and taurine-free; D4, 15% lipid and 1% taurine.

The positional distributions of key FAs in lipid molecules (TGs, PCs, and PEs) in the liver of fish fed with diets D3 and D4 are presented in Figure 7. To further investigate the distributions of six key FAs (such as 16:0, 18:1n-9, 18:2n-6, 20:4n-6, 20:5n-3, and 22:6n-3) in different lipid molecules, the percentages of different FAs in TGs, PCs, and PEs were calculated and compared.

The positional distributions of the key FAs, that is, 16:0, 18:1n-9, 18:2n-6, 20:4n-6, 20:5n-3, and 22:6n-3 were generally higher in TGs than in PCs and PEs. There were similar positional distributions of 18:1n-9 (*p* < 0.05), 18:2n-6 (*p* < 0.001), and 20:4n-6 (*p* < 0.01) in TGs, with higher contents of those FAs at the *sn-2* position than at the *sn-1* and *sn-3* positions for TGs fed with diets D3 and D4, while the positional distributions of 16:0 (*p* < 0.001) and 20:5n-3 (*p* < 0.01) were lower at the *sn-2* position than at the *sn-1* position for TGs. However, the contents of 22:6n-3 (*p* < 0.001) were higher at the *sn-3* position than at the *sn-2* position for TGs.

The positional distributions of both 16:0 and 18:1n-9 were higher (*p* < 0.05) at the *sn-1* position than at the *sn-2* position in PCs and PEs in fish fed with diets D3 and D4. 18:2n-6 showed the same trend in PEs but had the opposite trend in PCs. The positional distributions of 20:4n-6, 20:5n-3, and 22:6n-3 were higher (*p* < 0.05) at the *sn-2* position than at the *sn-1* position in PCs and PEs in fish fed with diet D3 and D4, except for 20:5n-3 attached at the *sn-1* position with higher (*p* < 0.05) positional distributions than at the *sn-2* position in PCs.

**FIGURE 8:**
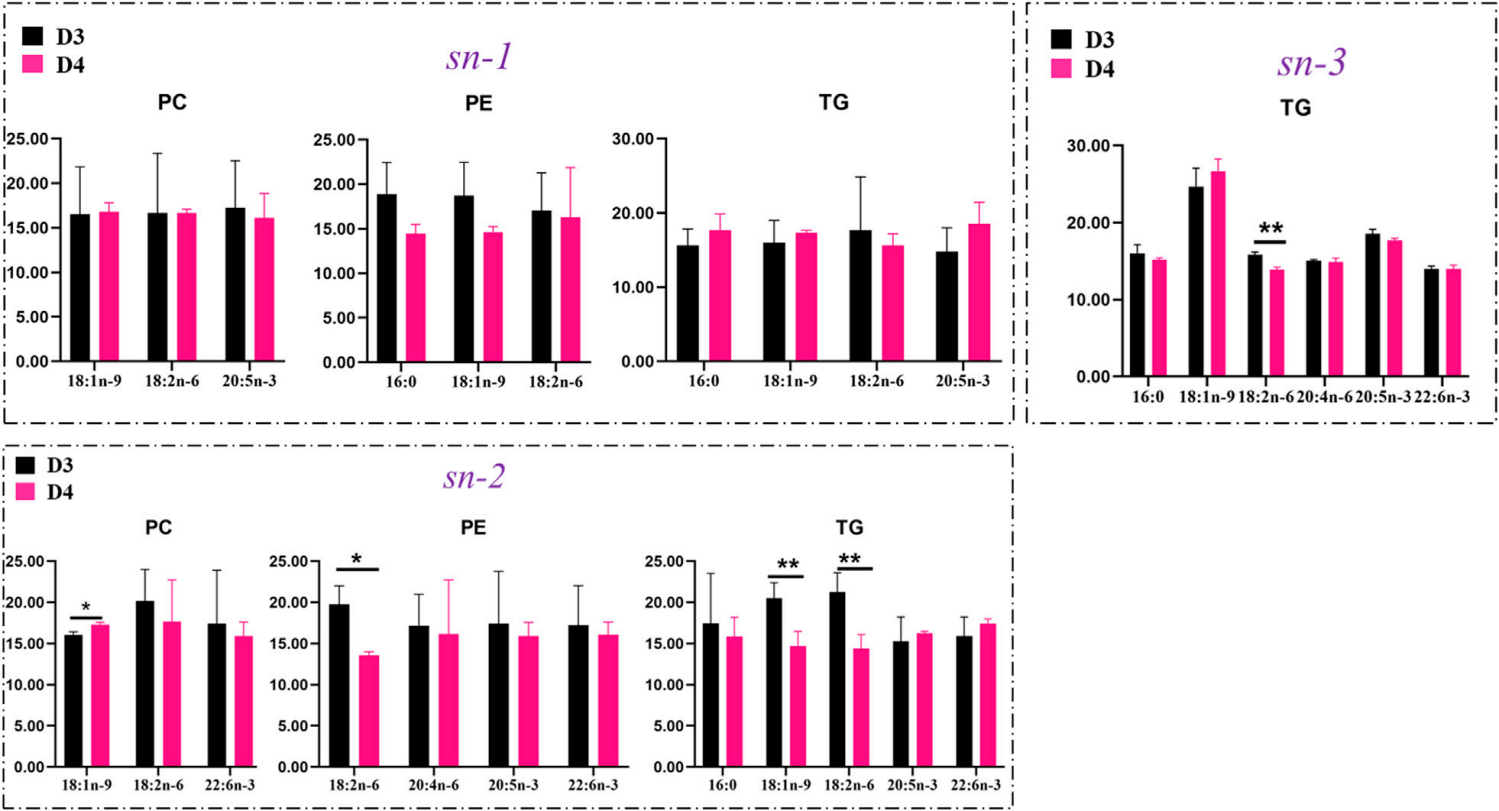
Comparison of contents of key fatty acids attached at the *sn-1*, *sn-2*, and *sn-3* positions in phosphatidylcholine (PC), phosphatidylethanolamine (PE), and triacylglycerol (TG) molecules in the liver of groupers fed with diet D3 and diet D4. The positions of fatty acid attachment on the glycerol backbone of triacylglycerol molecules are, respectively, named by stereospecific numbers, *sn-1*, *sn-2*, and *sn-3*, in sequence. Data are presented as the means of three triplicates per dietary treatment (mean ± SEM). Statistical analysis was performed using Student’s t-test. Asterisks (* and **) represent significant differences with *P* < 0.05 and *P* < 0.01, respectively. D3, 15% lipid and taurine-free; D4, 15% lipid and 1% taurine.

The positional distributions of key FAs in TGs, PCs, and PEs in the liver of fish fed with diet D4 vs. diet D3 are presented in Figure 8. Diet D4 decreased the contents of both 18:1n-9 and 18:2n-6 (*p* < 0.01) attached at the *sn-2* position in TGs and reduced contents of 18:2n-6 (*p* < 0.01) attached at the *sn-3* position and the contents of 18:2n-6 (*p* < 0.05) attached at the *sn-2* position in PEs but increased the contents of 18:1n-9 (*p* < 0.05) attached at the *sn-2* position in PCs, in comparison with diet D3.

## Discussion

Our current results showed that there was no significant difference in the growth rate between diet D1 (10% lipid and taurine-free) and diet D3 (15% lipid and taurine-free) lipid diets, indicating a tolerance to higher lipid diets within the lipid range applied (10–15% lipid). In this study, dietary taurine addition led to growth enhancement and feed utilization of grouper vs. taurine-free diets. This finding supported previous observations on other fish, such as yellowtail kingfish (*Seriola lalandi*) (Candebat et al., 2020) and meagre (*Argyrosomus regius*) (de Moura et al., 2018). The growth-promoting effect reflects a suitable nutritional status in the case of dietary taurine to meet the needs of fish. Furthermore, feeding diet D3 resulted in an increase in liver and muscle lipid contents of the grouper vs. feeding diet D1, suggesting that higher-lipid diets could improve lipid accumulation in this study and in previous studies on grass carp (*Ctenopharyngodon idella*) (Tang et al., 2019) and largemouth bass (*Micropterus salmoides*) (Zhou et al., 2020). In contrast, feeding the diets with taurine resulted in a decrease in liver lipid contents vs. feeding the diets without taurine in this study. The lipid-lowering effect of dietary taurine addition on the fish liver may be related to the regulation of taurine in the lipid metabolism (Koven et al., 2016; Garcia-Organista et al., 2019).

In the present study, 1% dietary taurine addition led to a decrease of lipid accumulation in the liver but not in the muscle. This finding reflected an inconsistent lipid accumulation in different tissues caused by dietary taurine (Koven et al., 2016). Here, we further determined lipid molecules to investigate the effect of dietary taurine on lipid accumulation in tissues of fish and its possible mechanism from the perspective of lipidomics. The results of hepatic lipidomics analysis showed that the hepatic abundance of TGs and PLs was upregulated by feeding diet D3 vs. diet D1. This may be the result of excessive lipid intake of fish (Liu et al., 2019). Compared to diet D1, dietary taurine addition resulted in 29 upregulated (27 PLs and 2 TGs) and 3 downregulated DLMs (2 TGs). Diet D4 (15% lipid and 1% taurine) reduced the liver content of all TGs but promoted the PL content compared to those of fish fed with diet D3. A previous study showed that TGs rather than PLs are the main form in the hepatocytes of Atlantic salmons (*Salmo salar*) (Stubhaug et al., 2005). Therefore, dietary taurine addition resulted in decreased liver contents of TGs and increased liver contents of PLs, indicating that the reduced liver lipid accumulation caused by dietary taurine addition may be associated with decreased TGs. The PLs are essential for the construction and renewal of cell membranes (Huang et al., 2021) due to their amphiphilic properties (Lordan et al., 2017). Their beneficial effects include the decrease in the absorption of cholesterol, the increase in plasma high-density lipoprotein cholesterol contents, better stability against oxidation (vs. TGs), and the metabolism involved in the rebalancing of inflammatory conditions, which reduces the risk of inflammatory disorders such as metabolic syndrome and diabetes (Lordan et al., 2017). The comparison results of liver lipid contents and DLMs between diet D2 and diet D1 and between diet D4 and diet D3 indicated that taurine addition could result in decreased liver lipid accumulation and maintain the liver cell function. Similar to our results, the increase of liver PL contents was accompanied with decreased TG contents in large yellow croakers (*Pseudosciaena crocea*) (Feng et al., 2017), hybrid snakeheads (*Channa maculata* ♀ × *Channa argus* ♂) (Lin et al., 2018), and tiger puffers (Xu et al., 2020). In the present study, the lipidomics analysis showed that the liver had more lipid molecules and DLMs than the muscle in the fish species; 15% lipid diets exhibited more DLMs than the 10% lipid diets. These results indicate a more pronounced response of lipid molecules to the dietary taurine content in the liver than that in the muscle in that the liver is the center of the lipid metabolism in fish.

Considering that the location of FAs on the glycerol backbone of lipid molecules is important in the utilization and hydrolysis of lipids and FAs (Wang et al., 2022; Xu et al., 2020), we further analyzed the compositions of predominant DLMs, that is, TGs, PCs, and PEs, in the liver of fish fed with diets D3 and D4. TGs in the liver accounted for the largest proportion of the DLMs by the comparison of D3 and D4, and the contents of 16:0, 18:1n-9, 18:2n-6, 20:4n-6, 20:5n-3, and 22:6n-3 were significantly higher in TGs than in PCs and PEs (Figure 7), indicating that TGs were the main class of lipid molecules in the accumulation of those FAs in the liver of groupers and previous observations in mud crabs (Wang et al., 2022), Nile tilapias (Liu et al., 2019), and Atlantic salmons (Stubhaug et al., 2005). TGs consist of a glycerol backbone esterified with three FAs at the *sn-1*, *sn-2*, and *sn-3* positions, while typical PLs, such as PCs and PEs, share a common structure consisting of two FAs esterified at the *sn-1/2* positions of the glycerol moiety, with phosphate and a base esterified to the *sn-3* position (Wang et al., 2022). It is generally accepted that the FAs attached at the *sn-2* position on the glycerol backbone are more stable than those attached at the *sn-1* or *sn-3* positions, which makes the former difficult to be hydrolyzed in the process of digestion and absorption (Liu et al., 2019; Wang et al., 2022). In our current study, the positional distributions of 18:1n-9, 18:2n-6, and 20:4n-6 at the *sn-2* position were significantly higher than that at the *sn-1* and *sn-3* positions of TGs in fish fed with diets D3 and D4, indicating that the *sn-2* position of TGs may be the preferential binding site for 18:1n-9, 18:2n-6, and 20:4n-6 in the liver of groupers. However, the positional distributions of 16:0 and 20:5n-3 were lower at position *sn-2* than at position *sn-1* in TGs. It was reported that an excess intake of lipids containing 16:0 at the *sn-2* position may increase the risk of atherogenesis, whereas FAs at the *sn-1* position are preferred for pancreatic lipase and are readily lipolyzed (Mattson and Volpenhein, 1962). 22:6n-3 is usually attached at the *sn-2* or *sn-3* positions in TGs, and it prefers position *sn-3* to position *sn-2*. Based on this fact, we could speculate that groupers receiving 15% lipid diets (diets D3 and D4) might preferentially deposit more 20:5n-3 and 22:6n-3 at the *sn-2* or *sn-3* positions of TGs than groupers receiving 10% lipid diets (diets D1 and D2). An analysis of TGs of the salmon muscle showed that 22:6n-3 located at the *sn-2* position was better retained than that located at the *sn-1/3* positions (Ruiz-Lopez et al., 2015). The positional distributions of 20:4n-6, 20:5n-3, and 22:6n-3 located at the *sn-1/3* positions in TGs in the Nile tilapia muscle could also allow these FAs to be easily hydrolyzed (Liu et al., 2019). A previous study reported that the excellent anti-inflammatory performance of seal oil came from the bonding preference of 20:5n-3 and 22:6n-3 for the *sn1/3* positions (Christensen et al., 1995).

The positional distribution of 18:2n-6 in liver lipid molecules of groupers has not been investigated before. The molecules of n-6 polyunsaturated FAs (PUFAs) mainly play a role in inducing the initiation and development of inflammation regarding a nutritional aspect. Thus, 18:2n-6 at the *sn-1* or *sn-3* positions of TGs might enhance the risks of inflammation (Liu et al., 2019). In the present study, compared to groupers fed with 10% lipid diets, fish fed with 15% lipid diets had higher contents of n-6 PUFAs in the liver, that is, TG (16:0/17:1/18:2) and TG (16:0/16:0/18:2) (Figure 5A and Supplementary Table S2) and in the muscle, that is, TG (14:0/22:4/22:4), TG (14:0/17:1/18:2), and TG (22:5/18:2/18:2) (Figure 5D and Supplementary Table S5). This indicated that a high dietary lipid content could lead to excessive accumulation of TGs containing 18:2n-6 in the liver and muscle, a potential risk of inflammation and atherogenesis. Diet D4 decreased the accumulation of 18:2n-6 at the *sn-2* or *3* positions of TGs, whereas diet D3 may be beneficial for the health status of fish when fed with 1% taurine diet (Figure 8). Our current study showed that dietary taurine addition may reduce liver lipid accumulation through decreasing the contents of TGs containing 18:2n-6 (Figure 5C and Figure 8). However, whether this finding will enhance the anti-inflammatory capacity of groupers remains to be further studied.

PCs are the most abundant PLs found in living cells, accounting for 40–50% of total cellular PLs, which play crucial roles in the regulation of membrane structures (Yang et al., 2018). PEs rank second, are particularly abundant in mitochondrial inner membranes, and contribute to the membrane structure (Yuan et al., 2021). Generally, saturated FAs (SFAs) and monounsaturated FAs (MUFAs) are preferentially esterified at position *sn-1* of PLs, while PUFAs are preferred to bonding position *sn-2* (Liu et al., 2019; Ruiz-Lopez et al., 2015; Tocher et al., 2008; Wang et al., 2022). The abovementioned results supported our current results, except for 18:2n-6 and 20:5n-3 located at the *sn-1* position in PEs and PCs, respectively (Figure 7). This finding may be due to the accumulation of more esterified PUFAs in lipid molecules such as TGs in groupers receiving 15% lipid diets vs. 10% lipid diets. Therefore, the lower contents of 18:2n-6 at the *sn-2* position of PEs in groupers fed with diet D4 may indicate a good health of groupers. MUFAs, especially 18:1n-9, are generally regarded as “healthy” FAs in human nutrition (Grundy, 1989). Diet D4 decreased 18:1n-9 accumulation at the *sn-2* position of TGs but enhanced its accumulation at the *sn-2* position of PCs in groupers vs. diet D3, indicating a different utilization of FAs in TGs and PCs because of preferential degradation of 18:1n-9 in TGs for energy supply than in PCs (Liu et al., 2019). However, the biological mechanisms underlying the different positional distributions of 18:1n-9 in various lipid classes are still unknown and need to be further studied (Liu et al., 2019). It was reported that PCs and PEs containing PUFA acyl groups at the *sn-2* position could enhance the fluidity of cell membranes (van der Veen et al., 2017). In this study, we observed that fish fed with diet D4 had lower contents of 18:2n-6 at the *sn-2/3* positions of TGs and higher contents of 18:1n-9 at the *sn-2* position of PCs vs. those fed with diet D3. This finding indicated that the liver lipid-lowering effect of dietary taurine not only helps to maintain the structural integrity of the fish cell membrane structure but also helps to improve their health status.

## Conclusion

In this study, dietary taurine led to both increased growth and feed utilization and a decreased liver lipid content in groupers fed with 10 and 15% lipid diets. The results of lipidomic analysis showed that the muscle was not as sensitive to varied dietary taurine contents as the liver with regard to lipid molecules. A significant reduction in the contents of all different TGs was caused by dietary taurine addition, and the opposite trend occurred for the contents of PLs. Irrespective of the diet, PUFAs were preferentially located at the *sn-2* position in PCs, while SFAs and MUFAs tended to be attached at the *sn-1* position of lipid molecules in the liver of groupers fed with 15% lipid diets vs. 10% lipid diets. The reducing liver lipid accumulation caused by dietary taurine addition may be through decreasing the contents of TGs containing 18:2n-6 at the *sn-2* and *sn-3* positions and, meanwhile, enhanced the anti-inflammatory capacity and health status of groupers. To the best of our knowledge, this is the first study to investigate the structures and changes of lipid molecular species in the liver of groupers in association with dietary taurine. This study will also provide a new insight into the function of taurine in farmed fish.

## Data Availability

The original contributions presented in the study are included in the article/Supplementary Materials; further inquiries can be directed to the corresponding author.
