# Supplementary material for: Lipidomic Profiling Reveals the Reducing Lipid Accumulation Effect of Dietary Taurine in Groupers (Epinephelus coioides)
Source: Front Mol Biosci. 2021 Dec 24;8:814318. doi: 10.3389/fmolb.2021.814318 (PMC8740052; doi:10.3389/fmolb.2021.814318)
Supplement: Supplementary file 2 [file Table1.DOCX]

**Table S1** Ingredients and proximate composition of experimental diets (on an as-fed basis, %).

| Ingredients | Diets containing 0 or 1% taurine and 10 or 15% lipid | | | |
| --- | --- | --- | --- | --- |
|  | D1 (0/10) | D2 (1/10) | D3 (0/15) | D4 (1/15) |
| Casein+gelation (4:1) | 50 | 50 | 50 | 50 |
| Corn starch | 24 | 24 | 24 | 24 |
| Blend oil (fish:soy oil=1:1) | 6 | 6 | 10 | 10 |
| Soy lecithin | 4 | 4 | 4 | 4 |
| Premix (vitamin:mineral=2:3) | 0.5 | 0.5 | 0.5 | 0.5 |
| Stay-C 35% | 0.02 | 0.02 | 0.02 | 0.02 |
| Ca(H_2_PO_4_)_2_ | 2 | 2 | 2 | 2 |
| Choline chloride | 0.3 | 0.3 | 0.3 | 0.3 |
| Shrimp meal | 4 | 4 | 4 | 4 |
| Microcrystalline cellulose | 7.18 | 6.18 | 3.18 | 2.18 |
| Sodium alginate | 2 | 2 | 2 | 2 |
| Taurine | 0 | 1 | 0 | 1 |
| Nutrient level |  |  |  |  |
| Dry matter | 91.2 | 91.4 | 90.2 | 90.3 |
| Crude protein | 46.6 | 47.0 | 46.8 | 46.6 |
| Crude lipid | 10.4 | 10.5 | 14.8 | 14.9 |
| Ash | 4.2 | 4.0 | 4.21 | 4.1 |
| Taurine | 0.04 | 0.96 | 0.04 | 0.98 |

All the ingredients were provided by Jiakang Feed Co. Ltd., Xiamen, China.

Vitamin premix (mg/kg feed): retinol acetate, 10; 1,25-dihydroxycholecalciferol, 10; DL-α-tocopherol acetate, 100; menadione sodium bisulfate, 10; thiamin nitrate, 10; riboflavin, 20; pyridoxine hydrochloride, 20; cyanocobalamin, 0.05; nicotinic acid, 50; calcium-*D*-pantothenate, 100; D-biotin, 1; *meso*-inositol, 500; folic acid, 4.

Mineral premix (mg/kg feed): ferric citrate, 497; CuSO_4_·5H_2_O, 24; ZnSO_4_·7H_2_O, 176; MnSO_4_·4H_2_O, 122; CoCl_2_·6H_2_O, 0.18; KIO_3_, 0.51; Na_2_SeO_3_, 0.33.
