# Supplementary material for: Lipidomic Profiling Reveals the Reducing Lipid Accumulation Effect of Dietary Taurine in Groupers (Epinephelus coioides)
Source: Front Mol Biosci. 2021 Dec 24;8:814318. doi: 10.3389/fmolb.2021.814318 (PMC8740052; doi:10.3389/fmolb.2021.814318)
Supplement: Supplementary file 5 [file Table2.DOCX]

**Table S2** Identified differential lipid molecules on pairwise comparison group (D1 vs D3, liver).

| LipidIon | IonFormula | CalMz | RT (min) | Fold Change | *P*-value | OPLS-DA VIP |
| --- | --- | --- | --- | --- | --- | --- |
| LPE(20:5)-H | C25 H41 O7 N1 P1 | 498.2626 | 1.9114 | 0.4750 | 0.02849 | 1.06 |
| LPE(20:4)-H | C25 H43 O7 N1 P1 | 500.2783 | 2.1628 | 0.3960 | 0.01049 | 1.15 |
| LPE(22:6)-H | C27 H43 O7 N1 P1 | 524.2783 | 2.0782 | 0.5579 | 0.01758 | 1.79 |
| LPC(22:6)+HCOO | C31 H51 O9 N1 P1 | 612.3307 | 2.0141 | 0.4309 | 0.02123 | 3.36 |
| PE(36:5p)-H | C41 H71 O7 N1 P1 | 720.4974 | 10.2837 | 0.4384 | 0.02541 | 2.62 |
| SM(d18:1/16:0)+HCOO | C40 H80 O8 N2 P1 | 747.5658 | 10.0119 | 0.6158 | 0.02949 | 1.26 |
| PE(18:0/20:3)-H | C43 H79 O8 N1 P1 | 768.5549 | 11.8666 | 0.5812 | 0.03557 | 1.69 |
| SM(d37:3)+HCOO | C43 H82 O8 N2 P1 | 785.5814 | 10.3897 | 0.7721 | 0.01483 | 1.05 |
| PE(18:1/22:5)-H | C45 H77 O8 N1 P1 | 790.5392 | 10.6105 | 0.5056 | 0.04621 | 1.06 |
| PC(16:0/18:1)+HCOO | C43 H83 O10 N1 P1 | 804.5760 | 11.1701 | 0.5364 | 0.04136 | 2.89 |
| PE(20:2/22:6)-H | C47 H77 O8 N1 P1 | 814.5392 | 10.4184 | 0.6120 | 0.02511 | 1.22 |
| PE(20:0/22:6)-H | C47 H81 O8 N1 P1 | 818.5705 | 11.1226 | 0.2551 | 0.00445 | 1.35 |
| PC(16:0/20:4)+HCOO | C45 H81 O10 N1 P1 | 826.5604 | 10.2301 | 0.3901 | 0.03997 | 2.50 |
| SM(d22:0/18:2)+HCOO | C46 H90 O8 N2 P1 | 829.6440 | 12.0299 | 0.6749 | 0.00422 | 1.39 |
| PC(18:0/18:1)+HCOO | C45 H87 O10 N1 P1 | 832.6073 | 12.1316 | 0.7570 | 0.02940 | 2.69 |
| PS(18:0/22:6)-H | C46 H77 O10 N1 P1 | 834.5291 | 10.0505 | 0.4410 | 0.04080 | 2.41 |
| PC(18:0/22:5)+HCOO | C49 H87 O10 N1 P1 | 880.6073 | 11.2624 | 0.7241 | 0.01648 | 1.24 |
| TG(16:0/16:0/18:3)+NH4 | C53 H100 O6 N1 | 846.7545 | 19.5303 | 0.9546 | 0.02914 | 3.29 |
| TG(16:0/17:1/18:2)+NH4 | C54 H102 O6 N1 | 860.7702 | 20.1540 | 0.6645 | 0.03736 | 3.79 |
| TG(16:0/18:3/20:4)+H | C57 H97 O6 | 877.7280 | 19.6630 | 1.9606 | 0.01300 | 2.52 |
| PC(44:12)+H | C52 H81 O8 N1 P1 | 878.5694 | 8.5744 | 0.3739 | 0.03791 | 1.05 |
| LPC(22:6)+H | C30 H51 O7 N1 P1 | 568.3398 | 2.0183 | 0.3525 | 0.04480 | 1.95 |

LipidIon, lipid molecule; IonFormula, lipid formula; CalMz, mass charge ratio; RT (min), retention time (min)

D1, 10% lipid and taurine-free; D3, 15% lipid and taurine-free.

**Table S3** Identified differential lipid molecules on pairwise comparison group (D1 vs D2, liver).

| LipidIon | IonFormula | CalMz | RT (min) | Fold Change | *P*-value | OPLS-DA VIP |
| --- | --- | --- | --- | --- | --- | --- |
| PE(36:5p)-H | C41 H71 O7 N1 P1 | 720.4974 | 10.2837 | 1.8867 | 0.00300 | 3.05 |
| PE(18:2/18:2)-H | C41 H73 O8 N1 P1 | 738.5079 | 10.4588 | 1.7321 | 0.04883 | 1.50 |
| PE(16:0/20:4)-H | C41 H73 O8 N1 P1 | 738.5079 | 9.9420 | 2.0141 | 0.01505 | 2.75 |
| PE(18:1p/20:5)-H | C43 H73 O7 N1 P1 | 746.5130 | 10.3609 | 1.8532 | 0.00261 | 2.46 |
| PG(16:0/18:1)-H | C40 H76 O10 N0 P1 | 747.5182 | 10.2836 | 2.1216 | 0.02450 | 2.79 |
| PE(18:0p/20:5)-H | C43 H75 O7 N1 P1 | 748.5287 | 11.3165 | 1.6661 | 0.03021 | 1.30 |
| PE(18:0/20:5)-H | C43 H75 O8 N1 P1 | 764.5236 | 10.8320 | 1.5791 | 0.04827 | 3.86 |
| PE(18:2p/22:6)-H | C45 H73 O7 N1 P1 | 770.5130 | 9.9457 | 1.7730 | 0.00880 | 2.18 |
| PG(18:1/18:2)-H | C42 H76 O10 N0 P1 | 771.5182 | 9.9522 | 1.7512 | 0.01135 | 1.54 |
| PE(18:1p/22:6)-H | C45 H75 O7 N1 P1 | 772.5287 | 10.7538 | 1.4524 | 0.01292 | 1.06 |
| PC(14:0/18:2)+HCOO | C41 H77 O10 N1 P1 | 774.5291 | 9.2773 | 1.8143 | 0.03034 | 1.11 |
| PE(17:1/22:2)-H | C44 H81 O8 N1 P1 | 782.5705 | 12.2018 | 2.8036 | 0.01315 | 1.06 |
| PS(18:0/18:2)-H | C42 H77 O10 N1 P1 | 786.5291 | 10.4956 | 1.5190 | 0.02876 | 1.05 |
| PC(16:0/18:2)+HCOO | C43 H81 O10 N1 P1 | 802.5604 | 10.4037 | 1.5447 | 0.01126 | 4.73 |
| PC(18:2/18:2)+HCOO | C45 H81 O10 N1 P1 | 826.5604 | 9.6621 | 1.6833 | 0.00322 | 3.99 |
| PC(18:1/18:2)+HCOO | C45 H83 O10 N1 P1 | 828.5760 | 10.4860 | 1.2543 | 0.03965 | 1.74 |
| PC(18:0/18:2)+HCOO | C45 H85 O10 N1 P1 | 830.5917 | 11.3841 | 1.5256 | 0.01921 | 3.00 |
| PG(20:0/20:0)-H | C46 H90 O10 N0 P1 | 833.6277 | 11.5989 | 1.9445 | 0.02725 | 6.71 |
| PC(19:0/18:2)+HCOO | C46 H87 O10 N1 P1 | 844.6073 | 11.8636 | 1.7829 | 0.01953 | 1.09 |
| PI(18:0/20:4)-H | C47 H82 O13 N0 P1 | 885.5499 | 10.1389 | 1.4877 | 0.03127 | 4.49 |
| PC(34:3)+H | C42 H79 O8 N1 P1 | 756.5538 | 9.6543 | 2.0296 | 0.00232 | 2.14 |
| PC(34:3)+H | C42 H79 O8 N1 P1 | 756.5538 | 9.4071 | 1.9162 | 0.00198 | 1.54 |
| PC(36:5)+H | C44 H79 O8 N1 P1 | 780.5538 | 8.8420 | 1.9465 | 0.03433 | 1.48 |
| PC(36:5)+H | C44 H79 O8 N1 P1 | 780.5538 | 9.4614 | 1.5241 | 0.01515 | 3.60 |
| PC(36:4)+H | C44 H81 O8 N1 P1 | 782.5694 | 9.6292 | 1.4521 | 0.04817 | 3.75 |
| PC(38:7)+H | C46 H79 O8 N1 P1 | 804.5538 | 8.9567 | 1.6851 | 0.02451 | 1.41 |
| PC(38:7)+H | C46 H79 O8 N1 P1 | 804.5538 | 8.6752 | 1.6559 | 0.02668 | 1.94 |
| PC(38:6)+H | C46 H81 O8 N1 P1 | 806.5694 | 9.5596 | 1.4537 | 0.04528 | 2.12 |
| Co(Q9)+NH4 | C54 H86 O4 N1 | 812.6551 | 16.1014 | 0.6157 | 0.03517 | 1.35 |
| TG(16:0/18:3/20:4)+H | C57 H97 O6 | 877.7280 | 19.6630 | 0.4871 | 0.00043 | 2.64 |
| TG(19:1/16:0/18:2)+NH4 | C56 H106 O6 N1 | 888.8015 | 20.1756 | 1.1710 | 0.02739 | 1.62 |
| PI(18:0/20:5)+NH4 | C47 H85 O13 N1 P1 | 902.5753 | 9.4337 | 1.5296 | 0.01553 | 1.01 |
| TG(20:1/18:1/22:5)+NH4 | C63 H112 O6 N1 | 978.8484 | 21.4003 | 0.6132 | 0.03749 | 2.11 |
| TG(18:1/18:2/24:1)+NH4 | C63 H118 O6 N1 | 984.8954 | 23.1511 | 1.6028 | 0.02525 | 1.51 |

LipidIon, lipid molecule; IonFormula, lipid formula; CalMz, mass charge ratio; RT (min), retention time (min)

D1, 10% lipid and taurine-free; D2, 10% lipid and 1% taurine.

**Table S4** Identified differential lipid molecules on pairwise comparison group (D3 vs D4, liver).

| LipidIon | IonFormula | CalMz | RT (min) | Fold Change | *P*-value | OPLS-DA VIP |
| --- | --- | --- | --- | --- | --- | --- |
| TG(16:0/20:5/20:5)+NH4 | C59 H98 O6 N1 | 916.7389 | 17.1302 | 0.1546 | 0.00002 | 1.38 |
| TG(20:1/18:1/22:5)+NH4 | C63 H112 O6 N1 | 978.8484 | 21.4003 | 0.1792 | 0.00003 | 2.06 |
| TG(18:1/20:5/22:6)+NH4 | C63 H102 O6 N1 | 968.7702 | 17.5624 | 0.1832 | 0.00006 | 1.78 |
| TG(18:3/18:2/22:6)+NH4 | C61 H100 O6 N1 | 942.7545 | 16.8727 | 0.1888 | 0.00009 | 1.83 |
| TG(18:3/18:2/18:3)+NH4 | C57 H98 O6 N1 | 892.7389 | 17.2764 | 0.2025 | 0.00012 | 2.20 |
| TG(22:5/18:2/22:6)+NH4 | C65 H104 O6 N1 | 994.7858 | 17.3981 | 0.2212 | 0.00013 | 1.26 |
| TG(18:3/18:2/22:6)+NH4 | C61 H100 O6 N1 | 942.7545 | 17.1499 | 0.1657 | 0.00014 | 1.35 |
| TG(18:2/22:6/22:6)+NH4 | C65 H102 O6 N1 | 992.7702 | 16.9389 | 0.1872 | 0.00014 | 1.34 |
| TG(18:1/18:2/20:4)+NH4 | C59 H104 O6 N1 | 922.7858 | 19.2651 | 0.2794 | 0.00016 | 4.16 |
| TG(18:3/18:2/22:5)+NH4 | C61 H102 O6 N1 | 944.7702 | 17.3152 | 0.1864 | 0.00025 | 1.47 |
| TG(22:5/18:2/22:5)+NH4 | C65 H106 O6 N1 | 996.8015 | 17.9558 | 0.2357 | 0.00027 | 1.11 |
| TG(18:3/14:1/18:2)+NH4 | C53 H94 O6 N1 | 840.7076 | 16.9329 | 0.2178 | 0.00027 | 1.33 |
| TG(20:5/18:2/18:2)+NH4 | C59 H100 O6 N1 | 918.7545 | 17.3883 | 0.2594 | 0.00029 | 3.31 |
| TG(16:0/20:5/22:6)+NH4 | C61 H100 O6 N1 | 942.7545 | 17.5318 | 0.2280 | 0.00030 | 1.59 |
| TG(16:0/14:0/16:0)+NH4 | C49 H98 O6 N1 | 796.7389 | 20.5220 | 0.3547 | 0.00030 | 1.69 |
| TG(18:0/16:0/20:0)+NH4 | C57 H114 O6 N1 | 908.8641 | 23.6948 | 0.3052 | 0.00038 | 1.06 |
| TG(16:0/18:3/22:6)+NH4 | C59 H100 O6 N1 | 918.7545 | 17.8608 | 0.2407 | 0.00043 | 2.22 |
| TG(16:0/18:1/23:0)+NH4 | C60 H118 O6 N1 | 948.8954 | 23.9313 | 0.2912 | 0.00049 | 1.22 |
| TG(16:0/16:0/16:0)+NH4 | C51 H102 O6 N1 | 824.7702 | 21.5987 | 0.3920 | 0.00052 | 1.83 |
| TG(22:5/18:2/20:4)+NH4 | C63 H104 O6 N1 | 970.7858 | 16.8752 | 0.2339 | 0.00063 | 1.11 |
| TG(18:1/20:4/22:6)+NH4 | C63 H104 O6 N1 | 970.7858 | 18.0135 | 0.2339 | 0.00068 | 1.73 |
| TG(18:3/18:2/20:5)+NH4 | C59 H98 O6 N1 | 916.7389 | 16.7525 | 0.2345 | 0.00079 | 1.42 |
| TG(18:2/17:1/22:6)+NH4 | C60 H102 O6 N1 | 932.7702 | 18.2966 | 0.2728 | 0.00086 | 1.11 |
| TG(22:5/18:2/18:2)+NH4 | C61 H104 O6 N1 | 946.7858 | 18.2542 | 0.2965 | 0.00088 | 2.65 |
| TG(16:0/22:6/22:6)+NH4 | C63 H102 O6 N1 | 968.7702 | 17.9334 | 0.2173 | 0.00089 | 1.61 |
| TG(18:3/18:2/18:3)+NH4 | C57 H98 O6 N1 | 892.7389 | 17.5518 | 0.2312 | 0.00091 | 2.68 |
| TG(18:1/18:2/22:5)+NH4 | C61 H106 O6 N1 | 948.8015 | 19.2872 | 0.2797 | 0.00100 | 3.21 |
| TG(18:1/20:2/22:5)+NH4 | C63 H110 O6 N1 | 976.8328 | 20.4188 | 0.3396 | 0.00101 | 1.37 |
| TG(16:0/18:1/24:0)+NH4 | C61 H120 O6 N1 | 962.9110 | 24.1595 | 0.3198 | 0.00103 | 1.57 |
| TG(18:0/20:5/22:6)+NH4 | C63 H104 O6 N1 | 970.7858 | 18.7863 | 0.2426 | 0.00108 | 1.27 |
| TG(18:3/18:2/20:5)+NH4 | C59 H98 O6 N1 | 916.7389 | 16.4859 | 0.2075 | 0.00119 | 1.35 |
| TG(18:2/18:2/22:6)+NH4 | C61 H102 O6 N1 | 944.7702 | 17.8135 | 0.2459 | 0.00121 | 3.89 |
| TG(20:5/18:2/22:6)+NH4 | C63 H100 O6 N1 | 966.7545 | 16.5322 | 0.2269 | 0.00151 | 1.23 |
| PC(40:7)+H | C48 H83 O8 N1 P1 | 832.5851 | 9.4967 | 1.8998 | 0.00154 | 1.44 |
| PC(16:1/20:5)+HCOO | C45 H77 O10 N1 P1 | 822.5291 | 8.7832 | 3.5868 | 0.00179 | 1.82 |
| TG(16:0/18:1/22:0)+NH4 | C59 H116 O6 N1 | 934.8797 | 23.6819 | 0.3573 | 0.00187 | 1.89 |
| PE(20:2/20:5)-H | C45 H75 O8 N1 P1 | 788.5236 | 10.2784 | 2.0279 | 0.00191 | 4.47 |
| TG(20:1/18:1/22:6)+NH4 | C63 H110 O6 N1 | 976.8328 | 21.0338 | 0.3425 | 0.00201 | 1.45 |
| TG(18:4/16:0/16:1)+NH4 | C53 H96 O6 N1 | 842.7232 | 18.2067 | 0.2859 | 0.00208 | 1.19 |
| TG(18:0/16:0/16:0)+NH4 | C53 H106 O6 N1 | 852.8015 | 22.4644 | 0.3670 | 0.00209 | 1.91 |
| TG(17:0/18:1/20:1)+NH4 | C58 H112 O6 N1 | 918.8484 | 22.8567 | 0.3048 | 0.00222 | 1.27 |
| TG(18:1/20:2/22:6)+NH4 | C63 H108 O6 N1 | 974.8171 | 19.9998 | 0.3211 | 0.00232 | 1.92 |
| TG(18:4/16:0/18:2)+NH4 | C55 H98 O6 N1 | 868.7389 | 18.2390 | 0.3056 | 0.00233 | 2.56 |
| TG(18:4/16:1/18:2)+NH4 | C55 H96 O6 N1 | 866.7232 | 17.1650 | 0.2072 | 0.00257 | 1.69 |
| TG(20:2/18:2/22:6)+NH4 | C63 H106 O6 N1 | 972.8015 | 18.9566 | 0.1949 | 0.00276 | 2.24 |
| TG(16:0/14:0/22:6)+NH4 | C55 H98 O6 N1 | 868.7389 | 18.6000 | 0.3340 | 0.00278 | 1.06 |
| TG(16:0/14:0/16:1)+NH4 | C49 H96 O6 N1 | 794.7232 | 19.3330 | 0.2983 | 0.00313 | 1.85 |
| TG(26:0/16:0/18:2)+NH4 | C63 H122 O6 N1 | 988.9267 | 24.1510 | 0.3802 | 0.00327 | 1.10 |
| TG(16:0/16:0/20:5)+NH4 | C55 H100 O6 N1 | 870.7545 | 19.4333 | 0.3523 | 0.00334 | 1.64 |
| TG(18:2/18:2/18:2)+NH4 | C57 H102 O6 N1 | 896.7702 | 19.4480 | 0.4020 | 0.00337 | 2.95 |
| PC(18:1/22:5)+HCOO | C49 H85 O10 N1 P1 | 878.5917 | 10.9509 | 1.5354 | 0.00362 | 1.09 |
| TG(18:3/18:2/18:2)+NH4 | C57 H100 O6 N1 | 894.7545 | 18.4062 | 0.3515 | 0.00376 | 4.09 |
| PC(18:2/22:6)+HCOO | C49 H81 O10 N1 P1 | 874.5604 | 9.1561 | 2.0967 | 0.00393 | 2.94 |
| TG(16:0/18:2/22:6)+NH4 | C59 H102 O6 N1 | 920.7702 | 18.8199 | 0.3617 | 0.00405 | 3.62 |
| TG(18:1/18:2/18:2)+NH4 | C57 H104 O6 N1 | 898.7858 | 20.3398 | 0.4281 | 0.00411 | 2.17 |
| TG(18:1/22:6/22:6)+NH4 | C65 H104 O6 N1 | 994.7858 | 17.9674 | 0.1563 | 0.00446 | 1.77 |
| TG(18:3/18:2/18:3)+NH4 | C57 H98 O6 N1 | 892.7389 | 16.8432 | 0.3585 | 0.00450 | 1.28 |
| TG(16:0/18:1/22:6)+NH4 | C59 H104 O6 N1 | 922.7858 | 19.8728 | 0.2854 | 0.00465 | 4.03 |
| TG(18:0/18:1/20:4)+NH4 | C59 H108 O6 N1 | 926.8171 | 21.4645 | 0.3537 | 0.00486 | 2.12 |
| TG(18:0/18:1/22:5)+NH4 | C61 H110 O6 N1 | 952.8328 | 21.4164 | 0.2082 | 0.00513 | 3.42 |
| PI(18:0/20:4)-H | C47 H82 O13 N0 P1 | 885.5499 | 10.1389 | 1.5559 | 0.00522 | 1.16 |
| TG(18:2/18:2/20:4)+NH4 | C59 H102 O6 N1 | 920.7702 | 18.0864 | 0.3254 | 0.00526 | 1.64 |
| TG(16:1/18:3/18:3)+NH4 | C55 H96 O6 N1 | 866.7232 | 16.7440 | 0.2930 | 0.00537 | 1.69 |
| TG(16:0/16:0/22:6)+NH4 | C57 H102 O6 N1 | 896.7702 | 19.8546 | 0.4028 | 0.00559 | 1.44 |
| TG(18:1/18:2/22:6)+NH4 | C61 H104 O6 N1 | 946.7858 | 18.8549 | 0.3051 | 0.00605 | 4.04 |
| TG(16:0/22:5/22:6)+NH4 | C63 H104 O6 N1 | 970.7858 | 18.4053 | 0.3173 | 0.00618 | 1.38 |
| TG(20:0/18:1/22:6)+NH4 | C63 H112 O6 N1 | 978.8484 | 21.9794 | 0.3278 | 0.00637 | 1.39 |
| PE(36:5p)-H | C41 H71 O7 N1 P1 | 720.4974 | 10.2837 | 2.0778 | 0.00638 | 2.05 |
| PC(20:5/18:2)+HCOO | C47 H79 O10 N1 P1 | 848.5447 | 8.7016 | 2.1141 | 0.00647 | 2.33 |
| TG(18:1/22:5/22:6)+NH4 | C65 H106 O6 N1 | 996.8015 | 18.4242 | 0.3166 | 0.00652 | 1.28 |
| TG(18:0/16:0/22:6)+NH4 | C59 H106 O6 N1 | 924.8015 | 21.0311 | 0.3505 | 0.00662 | 1.50 |
| TG(16:0/16:0/18:1)+NH4 | C53 H104 O6 N1 | 850.7858 | 21.6126 | 0.5418 | 0.00697 | 2.56 |
| TG(16:0/18:1/22:5)+NH4 | C59 H106 O6 N1 | 924.8015 | 20.3134 | 0.3091 | 0.00698 | 3.96 |
| TG(18:3/18:2/18:2)+NH4 | C57 H100 O6 N1 | 894.7545 | 17.7217 | 0.3904 | 0.00732 | 3.39 |
| TG(18:0/18:0/22:6)+NH4 | C61 H110 O6 N1 | 952.8328 | 22.0072 | 0.2172 | 0.00736 | 1.30 |
| TG(20:1/18:1/18:2)+NH4 | C59 H110 O6 N1 | 928.8328 | 21.7816 | 0.4356 | 0.00801 | 2.52 |
| TG(18:0/18:1/22:6)+NH4 | C61 H108 O6 N1 | 950.8171 | 21.0561 | 0.3514 | 0.00816 | 2.63 |
| TG(18:0/20:1/22:5)+NH4 | C63 H114 O6 N1 | 980.8641 | 22.2622 | 0.3498 | 0.00852 | 1.07 |
| TG(16:0/14:0/18:1)+NH4 | C51 H100 O6 N1 | 822.7545 | 20.5507 | 0.5132 | 0.00889 | 2.43 |
| TG(18:2/18:2/20:4)+NH4 | C59 H102 O6 N1 | 920.7702 | 18.4279 | 0.3743 | 0.00930 | 3.50 |
| PE(20:5/18:2)-H | C43 H71 O8 N1 P1 | 760.4923 | 8.9860 | 2.8421 | 0.00943 | 1.43 |
| PG(18:1/18:2)-H | C42 H76 O10 N0 P1 | 771.5182 | 9.9522 | 2.0264 | 0.00967 | 1.12 |
| TG(18:3/18:2/18:2)+NH4 | C57 H100 O6 N1 | 894.7545 | 18.0337 | 0.2939 | 0.01017 | 2.28 |
| PE(18:2p/22:6)-H | C45 H73 O7 N1 P1 | 770.5130 | 9.9457 | 2.0813 | 0.01074 | 1.61 |
| TG(18:1/18:2/18:3)+NH4 | C57 H102 O6 N1 | 896.7702 | 18.9868 | 0.3667 | 0.01276 | 3.39 |
| TG(16:0/18:2/18:3)+NH4 | C55 H100 O6 N1 | 870.7545 | 18.5486 | 0.4367 | 0.01287 | 3.65 |
| TG(18:1/22:5/22:6)+NH4 | C65 H106 O6 N1 | 996.8015 | 19.1422 | 0.1581 | 0.01328 | 1.47 |
| TG(18:1/18:2/18:3)+NH4 | C57 H102 O6 N1 | 896.7702 | 18.6801 | 0.4647 | 0.01377 | 4.38 |
| TG(56:6)+NH4 | C59 H106 O6 N1 | 924.8015 | 19.2970 | 0.2212 | 0.01417 | 2.29 |
| LPC(22:6)+H | C30 H51 O7 N1 P1 | 568.3398 | 2.0183 | 2.0727 | 0.01478 | 2.04 |
| TG(20:0/16:0/18:1)+NH4 | C57 H112 O6 N1 | 906.8484 | 23.1275 | 0.5246 | 0.01483 | 1.46 |
| PC(20:5/22:6)+HCOO | C51 H79 O10 N1 P1 | 896.5447 | 8.1440 | 2.0936 | 0.01515 | 1.07 |
| PS(39:4)-H | C45 H79 O10 N1 P1 | 824.5447 | 8.8650 | 2.3590 | 0.01546 | 1.86 |
| PC(16:1/22:6)+HCOO | C47 H79 O10 N1 P1 | 848.5447 | 8.9760 | 2.5632 | 0.01599 | 2.07 |
| PC(18:3/22:6)+HCOO | C49 H79 O10 N1 P1 | 872.5447 | 8.3461 | 2.2343 | 0.01664 | 1.18 |
| TG(14:0/14:0/18:2)+NH4 | C49 H94 O6 N1 | 792.7076 | 18.2596 | 0.4001 | 0.01826 | 1.07 |
| PE(18:1p/20:5)-H | C43 H73 O7 N1 P1 | 746.5130 | 10.3609 | 2.0879 | 0.01836 | 1.72 |
| TG(18:1/18:3/22:5)+NH4 | C61 H104 O6 N1 | 946.7858 | 17.3868 | 0.3778 | 0.01865 | 1.32 |
| TG(16:1/14:1/18:2)+NH4 | C51 H94 O6 N1 | 816.7076 | 17.3612 | 0.3586 | 0.01883 | 1.18 |
| PC(18:1/20:4)+HCOO | C47 H83 O10 N1 P1 | 852.5760 | 10.2585 | 1.7300 | 0.01943 | 1.40 |
| TG(18:0/16:0/18:1)+NH4 | C55 H108 O6 N1 | 878.8171 | 22.4596 | 0.5244 | 0.01985 | 2.09 |
| SM(d20:1/22:0)+HCOO | C48 H96 O8 N2 P1 | 859.6910 | 14.1622 | 2.1481 | 0.01991 | 1.23 |
| TG(18:1/18:1/22:6)+NH4 | C61 H106 O6 N1 | 948.8015 | 20.0460 | 0.2525 | 0.02085 | 4.39 |
| PE(18:2/22:6)-H | C45 H73 O8 N1 P1 | 786.5079 | 9.4335 | 1.7574 | 0.02088 | 1.93 |
| PC(18:1/22:6)+HCOO | C49 H83 O10 N1 P1 | 876.5760 | 10.0124 | 1.9699 | 0.02092 | 2.13 |
| PC(18:1/18:2)+HCOO | C45 H83 O10 N1 P1 | 828.5760 | 10.4860 | 1.2936 | 0.02123 | 2.43 |
| LPE(20:5)-H | C25 H41 O7 N1 P1 | 498.2626 | 1.9114 | 2.4918 | 0.02125 | 1.30 |
| TG(18:0/18:0/22:5)+NH4 | C61 H112 O6 N1 | 954.8484 | 22.2800 | 0.2954 | 0.02216 | 1.02 |
| PC(18:2/18:2)+HCOO | C45 H81 O10 N1 P1 | 826.5604 | 9.6621 | 1.8687 | 0.02332 | 2.30 |
| TG(16:1/18:2/18:3)+NH4 | C55 H98 O6 N1 | 868.7389 | 17.5996 | 0.4969 | 0.02343 | 2.24 |
| PC(39:7)+H | C47 H81 O8 N1 P1 | 818.5694 | 9.5465 | 2.0456 | 0.02398 | 1.15 |
| LPE(18:1)-H | C23 H45 O7 N1 P1 | 478.2939 | 2.9693 | 2.0281 | 0.02442 | 1.02 |
| PC(18:0/18:1)+HCOO | C45 H87 O10 N1 P1 | 832.6073 | 12.1316 | 1.2985 | 0.02461 | 1.60 |
| PG(20:0/20:0)-H | C46 H90 O10 N0 P1 | 833.6277 | 11.5989 | 2.0387 | 0.02595 | 4.18 |
| PE(16:0/20:4)-H | C41 H73 O8 N1 P1 | 738.5079 | 9.9420 | 2.5983 | 0.02615 | 2.27 |
| TG(18:3/17:1/18:2)+NH4 | C56 H100 O6 N1 | 882.7545 | 18.1895 | 0.4772 | 0.02648 | 1.09 |
| LPE(22:6)-H | C27 H43 O7 N1 P1 | 524.2783 | 2.0782 | 1.9338 | 0.02718 | 1.90 |
| TG(16:0/16:1/18:1)+NH4 | C53 H102 O6 N1 | 848.7702 | 20.5891 | 0.4552 | 0.02757 | 3.40 |
| PS(41:5)-H | C47 H81 O10 N1 P1 | 850.5604 | 9.5608 | 1.7198 | 0.02948 | 1.63 |
| PE(17:0/18:2)-H | C40 H75 O8 N1 P1 | 728.5236 | 11.1310 | 2.1792 | 0.02968 | 1.19 |
| PC(38:7)+H | C46 H79 O8 N1 P1 | 804.5538 | 8.6752 | 2.0578 | 0.02977 | 2.58 |
| LPE(18:2)-H | C23 H43 O7 N1 P1 | 476.2783 | 2.2385 | 1.9205 | 0.03332 | 1.28 |
| TG(52:4)+NH4 | C55 H102 O6 N1 | 872.7702 | 18.7600 | 0.3278 | 0.03352 | 3.01 |
| TG(16:0/18:1/20:1)+NH4 | C57 H110 O6 N1 | 904.8328 | 22.4366 | 0.5606 | 0.03406 | 2.34 |
| TG(18:0/16:0/18:0)+NH4 | C55 H110 O6 N1 | 880.8328 | 23.1386 | 0.3625 | 0.03433 | 1.33 |
| PG(20:0/22:5)-H | C48 H84 O10 N0 P1 | 851.5808 | 10.2461 | 2.2132 | 0.03613 | 2.05 |
| PE(18:2/20:4)-H | C43 H73 O8 N1 P1 | 762.5079 | 9.8724 | 1.9692 | 0.03653 | 1.46 |
| LPC(22:6)+HCOO | C31 H51 O9 N1 P1 | 612.3307 | 2.0141 | 1.9873 | 0.03739 | 1.92 |
| LPE(16:0)-H | C21 H43 O7 N1 P1 | 452.2783 | 2.8224 | 1.9736 | 0.03955 | 1.35 |
| TG(16:0/14:0/18:2)+NH4 | C51 H98 O6 N1 | 820.7389 | 19.4667 | 0.4899 | 0.04024 | 2.10 |
| TG(16:0/18:1/18:3)+NH4 | C55 H102 O6 N1 | 872.7702 | 19.6815 | 0.5888 | 0.04234 | 2.49 |

LipidIon, lipid molecule; IonFormula, lipid formula; CalMz, mass charge ratio; RT (min), retention time (min)

D3, 15% lipid and taurine-free; D4, 15% lipid and 1% taurine.

**Table S5** Identified differential lipid molecules on pairwise comparison group (D1 vs D3, muscle).

| LipidIon | IonFormula | CalMz | RT (min) | Fold Change | *P*-value | OPLS-DA VIP |
| --- | --- | --- | --- | --- | --- | --- |
| PC(18:0/20:5)+HCOO | C47 H83 O10 N1 P1 | 852.5760 | 10.6736 | 0.7819 | 0.02623 | 1.25 |
| PS(43:5)-H | C49 H85 O10 N1 P1 | 878.5917 | 11.0365 | 0.5655 | 0.01213 | 2.35 |
| PC(32:2)+H | C40 H77 O8 N1 P1 | 730.5381 | 9.3509 | 1.5399 | 0.01401 | 1.41 |
| TG(14:0/17:1/18:2)+NH4 | C52 H98 O6 N1 | 832.7389 | 18.9706 | 0.3332 | 0.03598 | 1.24 |
| TG(22:5/18:2/18:2)+NH4 | C61 H104 O6 N1 | 946.7858 | 17.5720 | 0.3883 | 0.04225 | 1.26 |
| TG(14:0/22:4/22:4)+NH4 | C61 H106 O6 N1 | 948.8015 | 18.6459 | 0.3609 | 0.04455 | 1.43 |
| PE(38:6p)-H | C43 H73 O7 N1 P1 | 746.5130 | 10.3788 | 0.4608 | 0.04741 | 1.54 |
| PC(16:0/16:0)+HCOO | C41 H81 O10 N1 P1 | 778.5604 | 11.1580 | 0.7119 | 0.04598 | 1.79 |
| PS(18:0/18:2)-H | C42 H77 O10 N1 P1 | 786.5291 | 10.5825 | 1.4841 | 0.01065 | 2.03 |
| PC(16:0e/18:2)+HCOO | C43 H83 O9 N1 P1 | 788.5811 | 11.1078 | 0.6371 | 0.02882 | 1.52 |
| PS(37:3)-H | C43 H77 O10 N1 P1 | 798.5291 | 8.7193 | 1.4283 | 0.00580 | 1.09 |
| PC(16:0e/20:4)+HCOO | C45 H83 O9 N1 P1 | 812.5811 | 10.9436 | 0.6313 | 0.00856 | 1.01 |

LipidIon, lipid molecule; IonFormula, lipid formula; CalMz, mass charge ratio; RT (min), retention time (min)

D1, 10% lipid and taurine-free; D3, 15% lipid and taurine-free.

**Table S6** Identified differential lipid molecules on pairwise comparison group (D1 vs D2, muscle).

| LipidIon | IonFormula | CalMz | RT (min) | Fold Change | *P*-value | OPLS-DA VIP |
| --- | --- | --- | --- | --- | --- | --- |
| PC(14:0e/18:1)+HCOO | C41 H81 O9 N1 P1 | 762.5654 | 10.8996 | 0.6514 | 0.01109 | 1.09 |

LipidIon, lipid molecule; IonFormula, lipid formula; CalMz, mass charge ratio; RT (min), retention time (min)

D1, 10% lipid and taurine-free; D2, 10% lipid and 1% taurine.

**Table S7** Identified differential lipid molecules on pairwise comparison group (D3 vs D4, muscle).

| LipidIon | IonFormula | CalMz | RT (min) | Fold Change | *P*-value | OPLS-DA VIP |
| --- | --- | --- | --- | --- | --- | --- |
| MGDG(41:11)-H | C50 H73 O10 | 833.5209 | 9.3103 | 1.5184 | 0.00261 | 1.45 |
| PC(16:0p/22:5)+HCOO | C47 H83 O9 N1 P1 | 836.5811 | 10.6300 | 1.2577 | 0.00542 | 2.24 |
| PI(18:0/22:6)-H | C49 H82 O13 N0 P1 | 909.5499 | 9.9516 | 1.1048 | 0.02595 | 1.95 |
| PC(36:4p)+H | C44 H81 O7 N1 P1 | 766.5745 | 10.2242 | 0.9067 | 0.02835 | 1.09 |
| PC(35:2)+H | C43 H83 O8 N1 P1 | 772.5851 | 10.9576 | 1.0692 | 0.01112 | 1.45 |
| PE(18:1/22:6)+H | C45 H77 O8 N1 P1 | 790.5381 | 10.3516 | 1.0830 | 0.02083 | 1.50 |
| PC(38:3)+H | C46 H87 O8 N1 P1 | 812.6164 | 11.4521 | 1.0834 | 0.04539 | 1.37 |
| TG(14:0/17:1/18:2)+NH4 | C52 H98 O6 N1 | 832.7389 | 18.9706 | 0.4441 | 0.03418 | 1.30 |
| PE(16:0/18:2)-H | C39 H73 O8 N1 P1 | 714.5079 | 10.7498 | 1.4544 | 0.01769 | 1.91 |
| PE(18:2/18:2)-H | C41 H73 O8 N1 P1 | 738.5079 | 10.0327 | 1.4022 | 0.01559 | 1.83 |
| PE(18:1/20:5)-H | C43 H73 O8 N1 P1 | 762.5079 | 9.9641 | 1.4553 | 0.00728 | 1.33 |
| PS(35:1)-H | C41 H77 O10 N1 P1 | 774.5291 | 9.3705 | 1.4485 | 0.01422 | 1.04 |
| PS(18:0/18:2)-H | C42 H77 O10 N1 P1 | 786.5291 | 10.5825 | 1.4453 | 0.02523 | 1.64 |
| PC(18:2/18:2)+HCOO | C45 H81 O10 N1 P1 | 826.5604 | 9.7521 | 1.2611 | 0.00330 | 4.33 |

LipidIon, lipid molecule; IonFormula, lipid formula; CalMz, mass charge ratio; RT (min), retention time (min)

D3, 15% lipid and taurine-free; D4, 15% lipid and 1% taurine.
