# Supplementary material for: Lipidomic Profiling Reveals the Reducing Lipid Accumulation Effect of Dietary Taurine in Groupers (Epinephelus coioides)
Source: Front Mol Biosci. 2021 Dec 24;8:814318. doi: 10.3389/fmolb.2021.814318 (PMC8740052; doi:10.3389/fmolb.2021.814318)
Supplement: Supplementary file 7 [file Table3.DOCX]

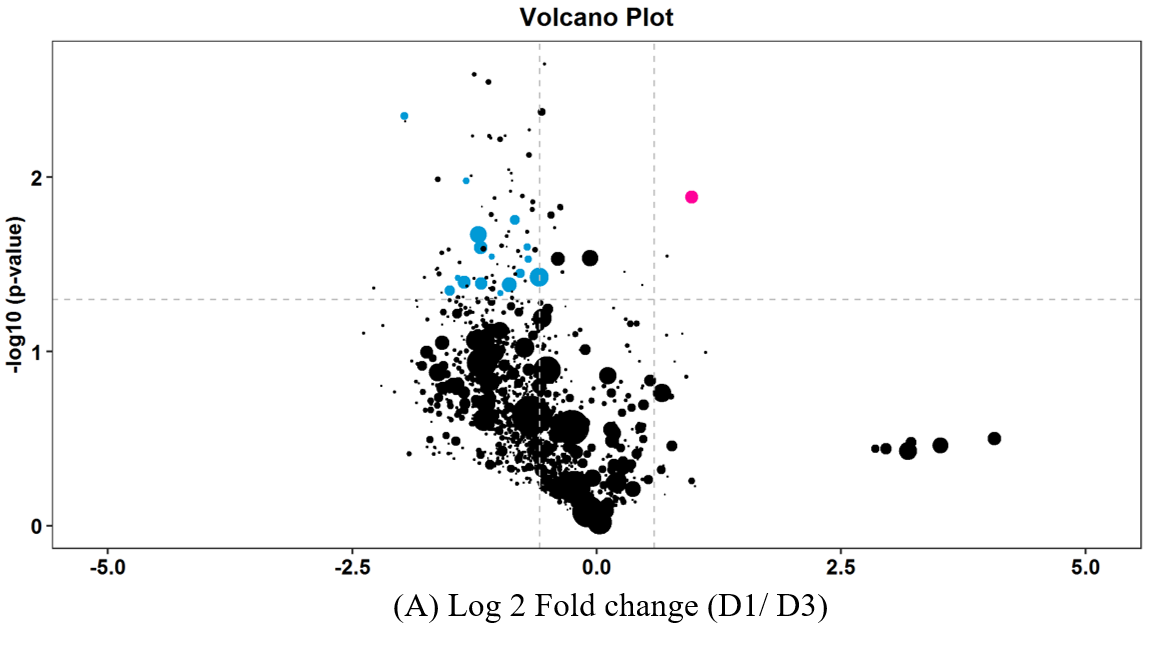

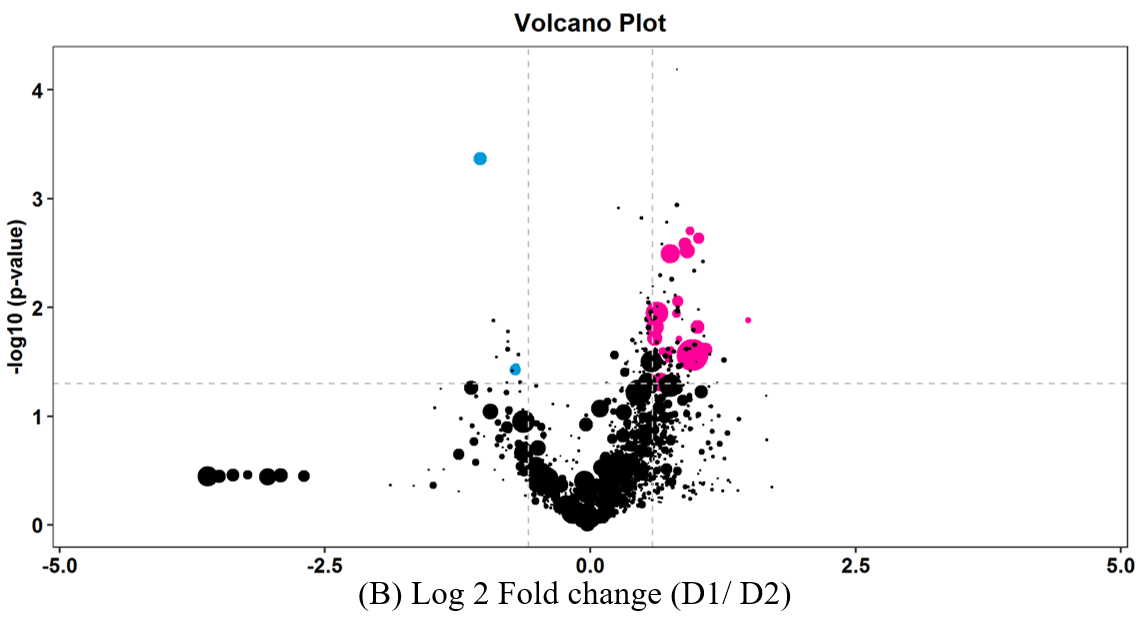

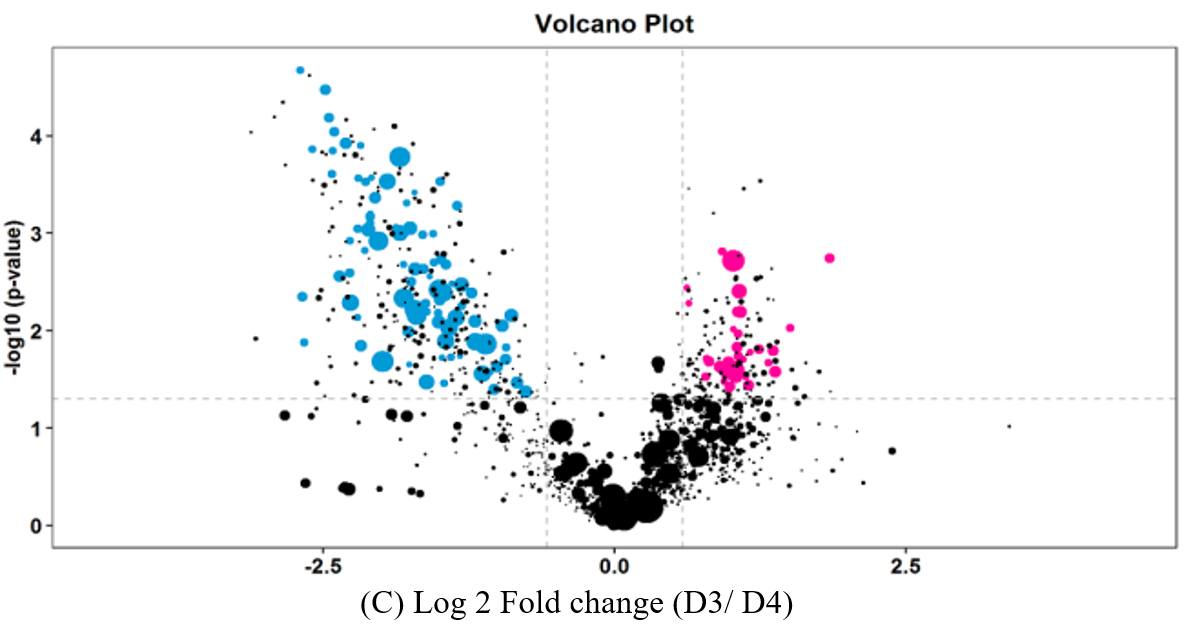


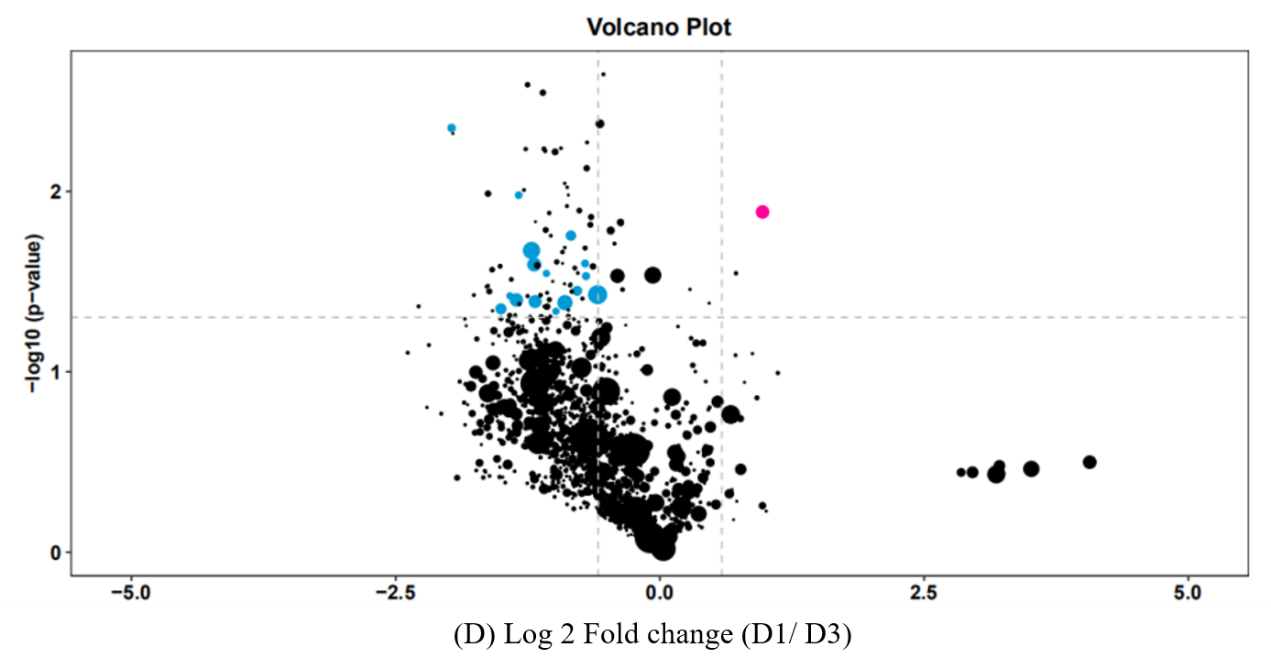


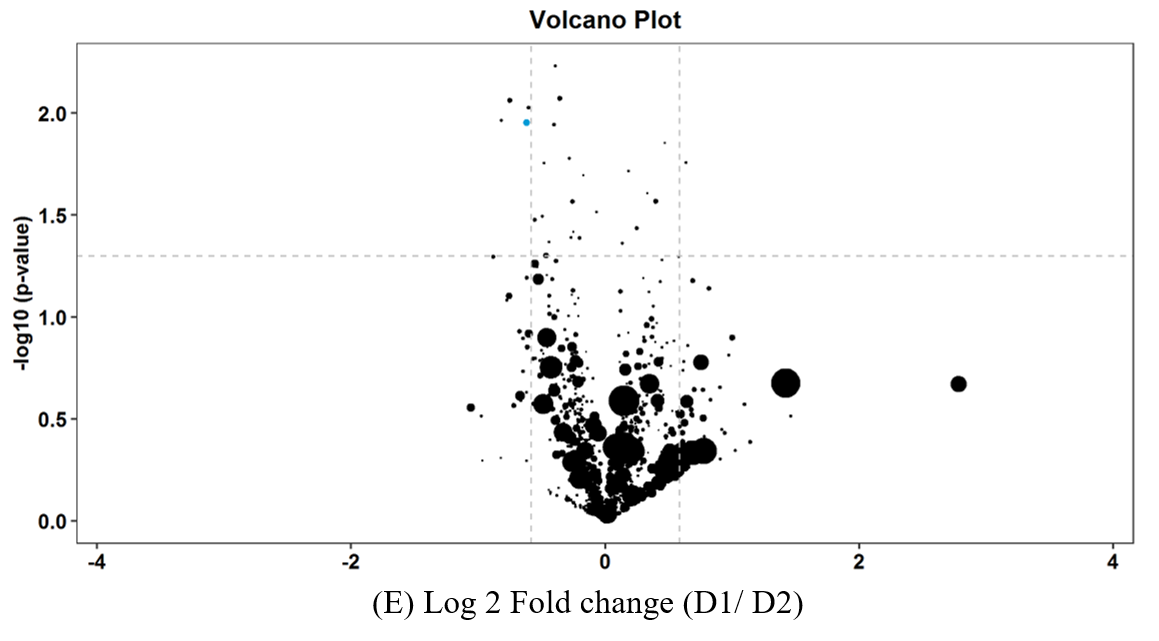


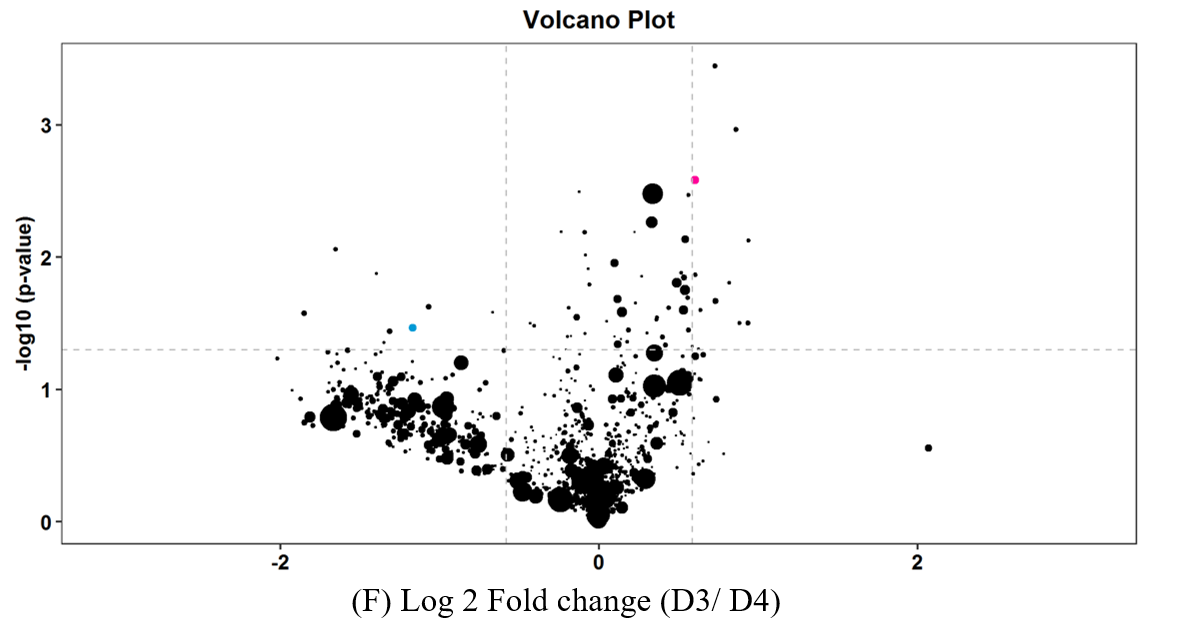


**Figure S1** Volcano plot of liver (**A-C**) and muscle (**D-F**) in different groups of groupers.

Excavating of differential lipid molecules in each pairwise comparison. Fig.4. Volcano plot showed the dots size represents the VIP value of the OPLS-DA model; the larger the VIP value, the larger the dot point. The red dot matches the condition (FC>1.5, P< 0.05, and VIP > 1.0), and blue dot match the condition (FC<0.67, P< 0.05, and VIP > 1.0). The differential lipid molecules labeled by the gray dot are not significant in a pairwise comparison.

D1, 10% lipid and taurine-free; D2, 10% lipid and 1% taurine; D3, 15% lipid and taurine-free; D4, 15% lipid and 1% taurine.
