# Supplementary material for: Lipidomic Profiling Reveals the Reducing Lipid Accumulation Effect of Dietary Taurine in Groupers (Epinephelus coioides)
Source: Front Mol Biosci. 2021 Dec 24;8:814318. doi: 10.3389/fmolb.2021.814318 (PMC8740052; doi:10.3389/fmolb.2021.814318)
Supplement: Supplementary file 8 [file Table4.DOCX]

Table S8 The fatty acid profiles (% of total fatty acids) in the liver of groupers fed diet D3 and diet D4 in a 56-d feeding period

| Fatty acid | D3 | D4 | *P* value |
| --- | --- | --- | --- |
| 14:0 | 0.69±0.11 | 0.28±0.07 | ** |
| 16:0 | 41.52±0.85 | 41.11±1.46 | NS |
| 18:0 | 15.10±0.67 | 19.71±1.60 | * |
| 20:0 | 1.74±0.22 | 2.55±0.15 | ** |
| 16:1n-7 | 2.16±0.08 | 1.89±0.08 | * |
| 18:1n-9 | 17.70±0.43 | 15.54±0.76 | * |
| 20:1n-9 | 4.89±0.19 | 5.95±0.83 | NS |
| 22:1n-11 | 0.33±0.03 | 0.31±0.05 | NS |
| 24:1 | 0.07±0.01 | 0.06±0.02 | NS |
| 18:2n-3 | 0.05±0.01 | 0.10±0.02 | ** |
| 18:2n-6 | 4.83±0.39 | 4.68±0.41 | NS |
| 20:2n-6 | 0.74±0.07 | 0.69±0.08 | NS |
| 18:3n-3 | 5.03±0.17 | 2.59±0.27 | *** |
| 18:4n-3 | 1.28±0.16 | 0.60±0.05 | ** |
| 20:4n-6 | 0.04±0.01 | 0.06±0.01 | NS |
| 22:5n-3 | 1.15±0.03 | 1.21±0.19 | NS |
| 22:6n-3 | 0.23±0.03 | 0.62±0.16 | * |

Data are presented as the means of three triplicates per dietary treatment (mean ± SEM).

Statistical analysis was performed using Student’s t-test.

NS, *, ** and *** represent significant differences with *P* > 0.05, *P* < 0.05, *P* < 0.01 and *P* < 0.001 respectively.

D3, 15% lipid and taurine-free; D4, 15% lipid and 1% taurine.
